# Supplementary material for: DISSeCT: An unsupervised framework for high-resolution mapping of rodent behavior using inertial sensors
Source: PLoS Biol. 2025 Oct 9;23(10):e3003431. doi: 10.1371/journal.pbio.3003431 (PMC12527166; doi:10.1371/journal.pbio.3003431)
Supplement: S1 Text — (PDF) [file pbio.3003431.s001.pdf]

DISSeCT: an unsupervised framework for high-resolution mapping of rodent behavior using inertial sensors

Supporting methods

Romain Fayat[1], Marie Sarraudy[1], Clément Léna[1], Daniela Popa[1],
Pierre Latouche[2] and Guillaume P. Dugué[1*]

[1]Institut de Biologie de l'École Normale Supérieure, École Normale Supérieure, CNRS, INSERM, Université PSL, Paris, France
[2]Laboratoire de Mathématiques Blaise Pascal, Institut Universitaire de France, Université Clermont Auvergne, CNRS, Aubière, France

[*]Corresponding author: guillaume.dugue@cnrs.fr

Contents

1 Change-point detection **3**
1.1 Principle and cost function . . . 3
1.2 Implementation . . . 3

2 Feature extraction **3**
2.1 Input data . . . 3
2.2 Per-segment spherical heading trajectories . . . 4
2.3 General statistics on inertial time series . . . 4
2.4 Gyroscope measurements in a gravitationally polarized reference frame . . . 5
2.5 Head tilt and 3D heading estimate . . . 5
2.6 Mean orientation and dispersion of head tilt and 3D heading . . . 5
2.7 Time-frequency features . . . 6

3 Automatic dimensionality reduction **6**
3.1 Implementation . . . 7

4 Clustering **7**
4.1 EM procedure . . . 8
4.1.1 E-Step . . . 8
4.1.2 M-Step . . . 8
4.2 Model selection . . . 8
4.3 Implementation . . . 9

5 Sequence analysis using a Hidden Markov Model with categorical emissions **9**
5.1 Model of the symbolic sequence . . . 9
5.2 Model parameters . . . 9
5.3 Priors and initialization . . . 10
5.4 EM procedure . . . 10
5.4.1 E-Step . . . 10
5.4.2 M-Step . . . 11
5.5 Hyperparameter optimization . . . 11

6 3D pose estimation **12**
6.1 Parameters for 2D pose estimate . . . 12
6.2 Frame loss . . . 12
6.3 Parameters for 3D pose estimation . . . 12
6.4 Obtaining a gravity-polarized Earth reference frame . . . 12

References **12**

1 Change-point detection

1.1 Principle and cost function

Given a time series $x = (x_1, \ldots, x_N)$ of length N, unsupervised change-point detection aims to find both the number K and the locations $\mathrm{T} = (\tau_k)_{k=1}^{K+1}$ of significant changes in the signal or its dynamics. For a given segmentation T, the total cost is defined as the sum over all segments of a function $c(\cdot)$ that quantifies the internal homogeneity of each segment, penalized by a term that increases with the number of segments K:

$$(\hat{\tau}_2, ..., \hat{\tau}_{\hat{K}}) = \arg\min_{(\tau_2,...,\tau_K),K} \sum_{k=1}^{K} c(x[\tau_k : \tau_{k+1}[) + \lambda K,$$

where $\tau_1 = 1$ and $\tau_{K+1} = N + 1$ are both fixed by convention. We used a Gaussian kernel-based RBF cost function c_{Gaussian}, which is sensitive to changes in the data distribution [1]. Using the kernel trick, the homogeneity cost of a segment $x[a : b[$ is computed as:

$$c_{\text{Gaussian}}(x[a : b[) = (b - a) - \frac{1}{b - a} \sum_{s,t=a}^{b-1} \exp(-\nu \parallel x_s - x_t \parallel^2).$$

The additive term $(b - a)$ arises from the general formulation of kernel-based cost rbf functions, where it represents the number of elements in the segment. While it does not affect the optimization outcome—since the total length of the time series is fixed across segmentations—it is conventionally included for mathematical completeness. In our implementation, this term has no impact on model selection or the detection of change-points.

The bandwidth parameter of the Gaussian kernel, ν, was set using the commonly applied median heuristic [1], based on a subset x_{selected} of 10,000 segments:

$$\nu = \frac{1}{\text{median}(x_{\text{selected}} \cdot x_{\text{selected}}^T)}.$$

Additionally, the minimum duration of the segments was set to 10 IMU samples (i.e., 33.3 ms for rat recordings), corresponding to one video frame to prevent spurious, very short segments from being detected by the algorithm.

1.2 Implementation

The implementation of PELT available in the ruptures package [2] was modified in order to perform the change-point detection pipeline on 1 min blocks independently. This modification allowed parallel change-points computation on the different subtime series and limited the space complexity of the computation ($\mathcal{O}(n^2)$ for the implementation in ruptures due to the computation of a full Gram matrix). Although this relaxed the guarantee of obtaining an optimal segmentation, the approximation had minimal impact on the output (S2C Fig).

2 Feature extraction

2.1 Input data

After obtaining the estimated change-points in IMU data $(\hat{\tau}_k)_{k\in[1,\hat{K}]}$, we obtain for each segment k among the $\hat{K}$:

- $\Omega_k = (\omega_{x,t} \quad \omega_{y,t} \quad \omega_{z,t})_{t\in[\hat{\tau}_k:\hat{\tau}_{k+1}[}$: angular speed measurement (°/s).
- $A_k = (a_{x,t} \quad a_{y,t} \quad a_{z,t})_{t\in[\hat{\tau}_k:\hat{\tau}_{k+1}[}$: acceleration measurement (g).

Writing sr the sampling rate, we also obtain:

- $duration_k = (\hat{\tau}_{k+1} - \hat{\tau}_k)/sr$: duration of the segment (s).

IMU data preprocessing (see Methods) yielded for each resulting segment:

- $Q_k = (q_t)_{t\in[\hat{\tau}_k:\hat{\tau}_{k+1}[} = (q_{w,t} \quad q_{x,t} \quad q_{y,t} \quad q_{z,t})_{t\in[\hat{\tau}_k:\hat{\tau}_{k+1}[}$: quaternion representing the orientation of the sensor relative to an arbitrary, gravity-polarized Earth reference frame.
- $A_{Gk} = (a_{Gx,t} \quad a_{Gy,t} \quad a_{Gz,t})_{t\in[\hat{\tau}_k:\hat{\tau}_{k+1}[} = Q_k^{-1}(0 \quad 0 \quad 1)$: estimate of the gravitational component of acceleration in the head reference frame, or head tilt.
- $A_{nGk} = Acc_k - Acc_{Gk}$: estimate of the non-gravitational component of acceleration in the head reference frame.

2.2 Per-segment spherical heading trajectories

Unit quaternions computed using an extended Kalman filter (see Methods) represent sensor orientation relative to an arbitrary, gravitationally polarized Earth reference frame and can therefore be considered as an estimate of the head's absolute orientation. While the tilt component of this estimate is highly reliable, as previously demonstrated [3], the azimuthal component (i.e., head direction in the Earth-horizontal plane) tends to drift due to gyroscope noise integration. Although magnetometer readings can be used to compensate for this drift, we chose to ignore them due to an inadequate magnetic environment. Nevertheless, magnetometer-free estimates of absolute head orientation can be used for short-duration segments, where azimuthal drift is minimal, particularly during rapid head movements characterized by a high gyroscope signal-to-noise ratio.

For each segment k, an estimate of the animal's absolute heading (corresponding to a spherical trajectory on the unit sphere) was computed by applying quaternions to the unit vector $(1 \quad 0 \quad 0)$ followed by a rotation about the Earth-vertical axis to align its first sample with an azimuth of zero:

$$Heading_k = R_k Q_k (1 \quad 0 \quad 0).$$

To obtain the rotation matrix R_k which performs the azimuthal alignment, the absolute orientation for the first sample of segment k is first computed:

$$heading_{Earth,\hat{\tau}_k} = q_{\hat{\tau}_k}(1 \quad 0 \quad 0) = (heading_{Earth,x,\hat{\tau}_k} \quad heading_{Earth,y,\hat{\tau}_k} \quad heading_{Earth,z,\hat{\tau}_k}).$$

From this initial absolute orientation, we then derive the initial azimuth $\theta_{\hat{\tau}_k}$ for segment k:

$$\theta_{\hat{\tau}_k} = arctan2(heading_{Earth,y,\hat{\tau}_k}, heading_{Earth,x,\hat{\tau}_k}).$$

Lastly, we define R_k as a rotation by an angle $-\theta_{\hat{\tau}_k}$ about the Earth-vertical axis:

$$R_k = \begin{pmatrix} cos\theta_{\hat{\tau}_k} & sin\theta_{\hat{\tau}_k} & 0 \\ -sin\theta_{\hat{\tau}_k} & cos\theta_{\hat{\tau}_k} & 0 \\ 0 & 0 & 1 \end{pmatrix}.$$

Aligning individual heading trajectories on the same initial azimuth allows overlaying them on the same graph to visualize head dynamics for multiple segments (see for example Fig 2D$_3$).

2.3 General statistics on inertial time series

For each segment k, the mean, standard deviation, minimum, maximum, median and quartiles were computed for each axis of Ω_k, A_{Gk} and A_{nGk}, as well as for their estimated gradients and the L_2 norm of Ω_k and A_{nGk}. This resulted in a total of 140 features.

We also calculated segment duration and its logarithm (base 10), adding two more features. All pairwise correlations between the axes of Ω_k and A_{nGk} were computed, resulting in an additional 15 features. Furthermore, the energy of gyroscope measurements and non-gravitational acceleration for each segment were included, adding 2 more features.

Additionally, we computed the number of zero-crossings in Ω_k, A_{Gk} and A_{nGk}, resulting in another 9 features.

2.4 Gyroscope measurements in a gravitationally polarized reference frame

Using our estimate of the head's absolute orientation, the coordinates of head angular velocity (measured by gyroscopes in the head reference frame) can be computed in an external, gravitationally polarized reference frame:

$$\Omega_{Ek} = (\omega_{Ex,t} \quad \omega_{Ey,t} \quad \omega_{Ez,t})_{t\in[\hat{\tau}_k:\hat{\tau}_{k+1}[} = Q_k gyr_k.$$

For each segment k, the component $(\omega_{Ez,t})_{t\in[\hat{\tau}_k:\hat{\tau}_{k+1}[}$ corresponds to the Earth-vertical (azimuthal) component of head angular velocity, i.e. its angular speed about the axis of gravity. This component, previously used to quantify circling behavior [3, 4], is here used to derive 9 features of particular interest for characterizing changes in head orientation:

- The mean, standard-deviation, minimum, maximum, median and quartiles of the cumulative change of azimuth over the segment, i.e. the cumulative sum of $(\omega_{Ez,t})_{t\in[\hat{\tau}_k:\hat{\tau}_{k+1}[}$ divided by the sampling rate.
- The total cumulative change of azimuth (net azimuthal change):

$$\frac{\sum_{t=\hat{\tau}_k}^{\hat{\tau}_{k+1}} \omega_{Ez,t}}{sr}.$$

- The net azimuthal speed:

$$\frac{\sum_{t=\hat{\tau}_k}^{\hat{\tau}_{k+1}} \omega_{Ez,t}}{\hat{\tau}_{k+1} - \hat{\tau}_k}.$$

2.5 Head tilt and 3D heading estimate

For each segment k, gravitational acceleration in the head reference frame:

$$A_{Gk} = a_{G,t\,t\in[\hat{\tau}_k:\hat{\tau}_{k+1}[} = (a_{Gx,t} \quad a_{Gy,t} \quad a_{Gz,t})_{t\in[\hat{\tau}_k:\hat{\tau}_{k+1}[}$$

was used to compute a total of 11 features:

- The coordinates of the initial (first sample) gravitational acceleration vector $a_{Gx,\hat{\tau}_k}$, $a_{Gy,\hat{\tau}_k}$ and $a_{Gz,\hat{\tau}_k}$
- The coordinates of the final (last sample) gravitational acceleration vector $a_{Gx,\hat{\tau}_{k+1}}$, $a_{Gy,\hat{\tau}_{k+1}}$ and $a_{Gz,\hat{\tau}_{k+1}}$
- The total change in the coordinates of the gravitational acceleration vector: $a_{Gx,\hat{\tau}_{k+1}-1} - a_{Gx,\hat{\tau}_k}$, $a_{Gy,\hat{\tau}_{k+1}-1} - a_{Gy,\hat{\tau}_k}$ and $a_{Gz,\hat{\tau}_{k+1}-1} - a_{Gz,\hat{\tau}_k}$
- The total change in head tilt (in degrees):

$$\arccos(a_{G,\hat{\tau}_k} \cdot a_{G,\hat{\tau}_{k+1}}).$$

- The net head tilt change speed (in °/s):

$$\frac{\arccos(a_{G,\hat{\tau}_k} \cdot a_{G,\hat{\tau}_{k+1}})}{duration_k}.$$

Similarly, the total change in 3D heading and net 3D heading change speed were computed from the first and last samples of the 3D heading estimate, totaling 2 features.

2.6 Mean orientation and dispersion of head tilt and 3D heading

Because head tilt and 3D heading follow trajectories on the unit sphere $\mathcal{S}^2$, their mean orientation and dispersion were estimated by approximating the mean and concentration of a von Mises–Fisher distribution [5].

Dispersion was calculated as the inverse square-root of the concentration. The resulting 8 features corresponded to the 3D coordinates of the mean orientation and the dispersion, for both head tilt and 3D heading.

2.7 Time-frequency features

The continuous wavelet transform of each axis of the angular velocity and non-gravitational acceleration time series was computed using the R package `WaveletComp`, with Morlet wavelets at dyadically spaced frequencies between 1 and 20 Hz (20 sub-octaves). The magnitude of wavelet coefficients were averaged into 7 linearly spaced bands between 2.5 and 20 Hz. For each segment, the base-10 logarithm of the median coefficients in these bands was taken, resulting in 42 additional features (7 frequency bands per axis).

3 Automatic dimensionality reduction

Principal component analysis (PCA) remains the most commonly used dimensionality reduction technique [6]. Unlike variable selection approaches, which focus on reducing the number of input variables of the original dataset, PCA builds a linear transformation of the data matrix to obtain a new data matrix characterized by fewer variables. By design, the new variables are all dependent on the original variables through this linear transformation. Introduced by Pearson [7] and rediscovered by Hotelling [8], PCA has been applied in numerous scientific fields across various applications. It can be described using geometrical properties or within an optimization framework. In the latter, considering $X \in \mathcal{M}_{N\times p}(\mathbb{R})$ as the original matrix of N observations (rows) and p quantitative variables (columns), and the linear transformation $Y = XU$ with $U \in \mathcal{M}_{p\times d}(\mathbb{R})$ and $d \leq p$, PCA can be framed through the following maximization problem:

$$\begin{aligned}\hat{U} &= \underset{U}{\operatorname{argmax}} \operatorname{Tr}\left(\frac{Y^\intercal Y}{N}\right)\\ &= \underset{U}{\operatorname{argmax}} \operatorname{Tr}\left(U^\intercal \frac{X^\intercal X}{N} U\right)\\ &= \underset{U}{\operatorname{argmax}} \sum_{j=1}^{d} U_j^\intercal \frac{X^\intercal X}{N} U_j,\end{aligned} \tag{1}$$

under the constraint that $U^\intercal U = I_d$. In Equation (1), Tr denotes the trace operator which sums the diagonal elements of a square matrix and $U_j \in \mathbb{R}^p$ is column j of matrix U. By construction, if the dataset in X is centered (if not, a centering operation is employed), $X^\intercal X/N = C_X$ is the empirical covariance matrix associated with the data in the original matrix X. To limit the influence of variables with large variances in X on the construction of U in PCA, the variables in X are almost always scaled so that C_X becomes the empirical correlation matrix. Using spectral theory arguments, it can be shown that the optimal solution to this problem is obtained through an eigendecomposition of C_X [9]. Thus, column $\hat{U}_1$ of matrix $\hat{U}$ is set to the eigenvector of C_X associated with the largest eigenvalue λ_1, $\hat{U}_2$ to the one with the second largest, and so on up to $\hat{U}_d$. We emphasize that since X is centered and $Y = X\hat{U}$, so is Y. Therefore, $Y^\intercal Y/N = C_Y = \hat{U}^\intercal C_X \hat{U} = \operatorname{diag}(\lambda_1, \lambda_2, \ldots, \lambda_d)$ is the empirical covariance matrix with entries λ_j on the diagonal and 0 elsewhere. So, the new d variables created in matrix Y have no correlation. Moreover, since the goal is to maximize $\operatorname{Tr}(Y^\intercal Y/N) = \operatorname{Tr}(C_Y)$ in Equation (1), it can be emphasized that the key objective at the core of PCA is to build a new dataset $Y \in \mathcal{M}_{N\times d}(\mathbb{R})$ from X with less variables so that the new observations in Y have maximal spread (maximal variance), with uncorrelated variables. Besides, at the optimal matrix $\hat{U}$, the objective function becomes:

$$\sum_{j=1}^{d} \hat{U}_j^\intercal C_X \hat{U}_j = \sum_{j=1}^{d} \lambda_j.$$

If d is set to p, corresponding to the case where all dimensions are preserved, then

$$\begin{aligned}\mathrm{Tr}(C_Y) &= \sum_{j=1}^{p} \lambda_j \\ &= \mathrm{Tr}(C_X),\end{aligned}$$

so that the sum of the empirical variances of the original dataset X are exactly preserved in the new dataset Y. In practice, d is chosen to be small compared to p and the percentage of variance of X kept in Y is $100 \sum_{j=1}^{d} \lambda_j / \sum_{j'=1}^{p} \lambda'_j$.

In spite of the popularity of PCA, no authoritative solution exists for choosing the number d of eigenvectors (principal components, PCs) in U to build Y. Most existing applications choose the dimension d by considering the scree plot of eigenvalues of C_X. This ad-hoc technique, popularized by Cattell [10], has been largely modified and perfected over the last 50 years [11]. Recently, focusing on the probabilistic version of PCA [12], an analytical expression of the marginal likelihood was obtained, allowing d to be estimated [11, 13]. In this paper, we rely on the cross-validation approximation strategy of Josse and Husson [14] which scales well with larger data matrices.

3.1 Implementation

We used the package `scikit-learn` in Python to perform PCA with the empirical correlation matrix of the data. The number of dimension d was estimated using the cross-validation approximation strategy of Josse and Husson [14], using the function *estim_ncp* from the package *FactoMineR* in R.

4 Clustering

In this paper, we relied on the expectation maximization (EM) algorithm [15] for GMMs which is widely seen as a reference for clustering [16]. Unlike k-means [17], for instance, which assumes the clusters of observations to be characterized by spheres in the space of variables, fewer assumptions are made in GMMs such that clusters with different shapes can be uncovered. In particular, GMMs allow both spheres as well as ellipsoids with different volumes, shapes, and directions, to be handled. Moreover, contrary to k-means, no assumption is made on the average size that the clusters of observations should have [18]. Finally, it is a soft clustering approach based on a probabilistic model. As such, it provides statistics, which strongly help interpreting the clusters found, in practice, and it gives access to theoretically validated mathematical tools from computational statistics [19]. In particular, contrary to most clustering approaches which rely on heuristics to estimate the number of clusters present in the data, model selection criteria with theoretical guarantees can be used [20].

Denoting $X \in \mathcal{M}_{N \times p}(\mathbb{R})$ the matrix of N observations and p quantitative variables with row i, $x_i \in \mathbb{R}^p$, the GMM model considers the following mixture density function with K components:

$$p(x|\pi, \theta) = \sum_{k=1}^{K} \pi_k \mathcal{N}(x; \mu_k, \Sigma_k),$$

where $\mathcal{N}(x; \mu_k, \Sigma_k)$ is the density function of a multidimensional Gaussian law with mean μ_k and covariance matrix Σ_k evaluated at x. We note $\theta = \{(\mu_1, \Sigma_1), \ldots, (\mu_K, \Sigma_K)\}$ the set of all parameters controlling the Gaussian density functions. Moreover, $\pi \in [0, 1]^K$ is a vector of mixing weights which are probabilities such that $\sum_{k=1}^{K} \pi_k = 1$. The log-likelihood of the model associated with the observations in X is given by:

$$\log p(X|\pi, \theta) = \sum_{i=1}^{N} \log(\sum_{k=1}^{K} \pi_k \mathcal{N}(x_i; \mu_k, \Sigma_k)). \quad (2)$$

Unfortunately, maximizing Equation (2) does not lead to analytic expressions for π and θ. The EM algorithm [15] was introduced in 1977 to tackle this issue and has been widely used as a solution to this problem

since then.

4.1 EM procedure

The EM algorithm for fitting a GMM involves two main steps as well as an initialization.

Initialization

The EM algorithm is initialized with a k-means algorithm with K clusters applied on X, such that τ_{ik} is set to 1 if observation i was found in cluster k, 0 otherwise.

- **E Step:** Calculate the expected posterior values τ_{ik} that observation i is actually from cluster k, using the current estimates of the model parameters (π, θ)
- **M Step:** Calculate the estimators for π and θ, using the current estimate of the τ_{ik}

The E and M steps are repeated in a loop which maximizes the log-likelihood of the data. The loop is stopped when (2) converges.

4.1.1 E-Step

Calculate

$$\tau_{ik} = \frac{\pi_k \mathcal{N}(x_i; \mu_k, \Sigma_k)}{\sum_{l=1}^{K} \pi_l \mathcal{N}(x_i; \mu_l, \Sigma_l)}, \forall i \in \{1, \ldots, N\}, k \in \{1, \ldots, K\}.$$

4.1.2 M-Step

Calculate

$$\pi_k = \frac{\sum_{i=1}^{N} \tau_{ik}}{N}, \forall k \in \{1, \ldots, K\},$$

$$\mu_k = \frac{\sum_{i=1}^{N} \tau_{ik} x_i}{\sum_{i=1}^{N} \tau_{ik}},$$

and

$$\Sigma_k = \frac{\sum_{i=1}^{N} \tau_{ik} (x_i - \mu_k)(x_i - \mu_k)^\intercal}{\sum_{i=1}^{N} \tau_{ik}}.$$

4.2 Model selection

In this paper, we relied on the BIC criterion for GMM [16] to estimate the number of clusters present in the data matrix X. It is given by:

$$BIC(K) = 2 \log p(X|\hat{\pi}, \hat{\theta}) - m_K \log N, \quad (3)$$

where $\hat{\pi}$ and $\hat{\theta}$ are the estimators found by the EM algorithm for K clusters, and m_K is the total number of free parameters. For a full GMM model without constraints on the covariance matrices Σ_k, m_K is given by:

$$m_K = K - 1 + Kp + K\frac{p(p+1)}{2}.$$

In practice, we considered a grid from 5 to 100 for K, and for each value, a full EM algorithm was employed to compute $BIC(K)$. Moreover, several types of covariance structures (full, diagonal, tied, spherical) corresponding to assumptions on the shapes of the clusters were tested. Finally, the model retained was the one for which the number of clusters and the type of covariance structure were maximizing the BIC criterion over the grid search.

4.3 Implementation

We used the package `scikit-learn` in Python to perform BIC and EM for GMM on the data matrix. Since EM is only guaranteed to reach a local maximum of the log-likelihood (2), we repeated the whole procedure 10 times for multiple initialization.

5 Sequence analysis using a Hidden Markov Model with categorical emissions

Following the application of DISSeCT to the inertial data, we obtained N segments, each assigned to one of $K-1$ clusters identified by the GMM with the highest posterior probability, alongside an additional label for outliers. This process resulted in a symbolic sequence representing the clusters.

To capture the temporal dynamics of this sequence, we employed a categorical hidden Markov model (HMM) with $\mathcal{S}$ hidden states, utilizing the `hmmlearn` library. The model parameters, including transition and emission probabilities, were estimated using the EM algorithm, as detailed in the following sections.

5.1 Model of the symbolic sequence

The symbolic sequence of K possible clusters associated with each of the N segments, denoted by Y, was modeled as resulting from a sequence of hidden states S. The HMM used to represent this sequence is defined as follows:

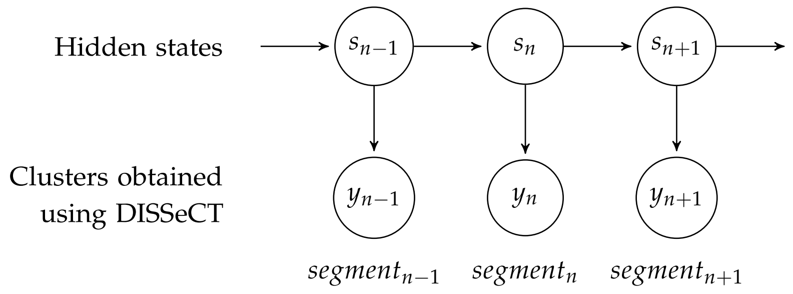

HMM graphical model of the symbolic sequence of clusters associated with individual segments.

In the following, we denote:

$$Y = y_1 y_2 \dots y_N \in [\![1, K]\!]^N,$$

and

$$S = s_1 s_2 \dots s_N \in [\![1, \mathcal{S}]\!]^N,$$

where Y is the observed sequence of clusters, and S is the corresponding sequence of hidden states.

5.2 Model parameters

The model parameters encompass:

- **Initial state distribution** $\pi = (\pi_i)_{i \in [\![1,\mathcal{S}]\!]}$:

$$\pi_i = \mathbb{P}(s_1 = i),$$

- **Transition matrix** $A = (a_{ij})_{i,j \in [\![1,\mathcal{S}]\!]^2}$:

$$a_{ij} = \mathbb{P}(s_{n+1} = j | s_n = i),$$

- **Emission matrix** $E = (e_{ik})_{i \in [\![1,\mathcal{S}]\!], k \in [\![1,K]\!]}$:

$$e_{ik} = \mathbb{P}(y_n = k | s_n = i).$$

Under the categorical HMM, the likelihood of the observed data Y, given the model parameters $\theta = (\pi, A, E)$, is expressed as:

$$p(Y|\theta) = \sum_S \pi_{s_1} e_{s_1 y_1} \prod_{n=2}^{N} a_{s_{n-1} s_n} e_{s_n y_n}.$$

This likelihood represents the probability of observing the sequence Y by summing over all possible sequences of hidden states S.

5.3 Priors and initialization

We initialize the parameters of the model $\theta = (\pi, A, E)$ as follows:

- **Initial state distribution** π**:** The initial state distribution π is initialized using a Dirichlet prior distribution. Thus, each state $i \in \{1, \ldots, \mathcal{S}\}$ is assigned an initial probability drawn from a Dirichlet distribution, with a parameter vector $\alpha_\pi = (\frac{1}{\mathcal{S}}, \frac{1}{\mathcal{S}}, \ldots, \frac{1}{\mathcal{S}})$:

$$\pi \sim \text{Dirichlet}(\alpha_\pi).$$

- **Transition matrix** A**:** The transition probabilities A are also initialized using a Dirichlet prior distribution. Thus, for each state $i \in \{1, \ldots, \mathcal{S}\}$, the transition probabilities to all other states are drawn from a Dirichlet distribution with parameter vector $\alpha_A = (\frac{1}{\mathcal{S}}, \frac{1}{\mathcal{S}}, \ldots, \frac{1}{\mathcal{S}})$:

$$a_{i.} \sim \text{Dirichlet}(\alpha_A),$$

  where $a_{i.}$ denotes row i of matrix A and is the set of all transition probabilities from state i.
- **Emission matrix** E**:** The emission probabilities E are initialized uniformly. Thus, each element e_{ik} is first drawn from a continuous uniform distribution between 0 and 1:

$$e_{ik} \sim \text{Uniform}(0, 1).$$

  Then, the rows are normalized to ensure they sum to 1. Thus, for each state $i \in \{1, \ldots, \mathcal{S}\}$:

$$e_{ik} = \frac{e_{ik}}{\sum_{k'=1}^{K} e_{ik'}}.$$

5.4 EM procedure

The EM algorithm for fitting a categorical HMM involves two main steps:

- **E Step:** Calculate the expected value of the complete log-likelihood function, using the current estimates of the model parameters.
- **M Step:** Maximize this expected complete log-likelihood function to update the model parameters.

The procedure was repeated for 100 iterations to ensure that the model parameters are optimized to best fit the observed data Y.

5.4.1 E-Step

In the E step, we utilize the Viterbi filter to determine the most likely sequence of hidden states, given the observed data Y and the current estimates of the parameters.

1. **Initialization:** Initialize the Viterbi variables and the back-pointer array ψ:

$$\delta_1(i) = \pi_i e_{iy_1} \quad \text{for } i \in \{1, \ldots, \mathcal{S}\},$$

$$\psi_1(i) = 0 \quad \text{for } i \in \{1, \ldots, \mathcal{S}\}.$$

2. **Recursion:** For each time step $n = 2, \ldots, N$ and for each state $j \in \{1, \ldots, \mathcal{S}\}$:

$$\delta_n(j) = \max_{i \in \{1,\ldots,\mathcal{S}\}} [\delta_{n-1}(i) a_{ij}] \cdot e_{jy_n},$$

$$\psi_n(j) = \arg\max_{i \in \{1,\ldots,\mathcal{S}\}} [\delta_{n-1}(i) a_{ij}].$$

3. **Termination:** Find the highest probability of the final state:

$$P^* = \max_{i \in \{1,\ldots,\mathcal{S}\}} \delta_N(i).$$

4. **Path Backtracking:** Backtrack through the computed variables to find the most probable sequence of hidden states. Start with the state that has the highest probability at the final time step:

$$s_N^* = \arg\max_{i \in \{1,\ldots,\mathcal{S}\}} \delta_N(i).$$

For $n = N-1, N-2, \ldots, 1$, determine the preceding state using the back-pointers, which track the state transitions:

$$s_n^* = \psi_{n+1}(s_{n+1}^*).$$

Here, ψ is an array of back-pointers that stores the indices of the states that led to the maximum probability at each time step, facilitating the reconstruction of the most likely state sequence.

5.4.2 M-Step

In the M-step, we update the model parameters $\theta = (\pi, A, E)$ based on the state sequence $S^* = s_1^* s_2^* \ldots s_N^*$ obtained from the E-step.

1. **Update initial probabilities:**

$$\pi_i = \frac{\delta_{s_1^*}(i)}{\sum_{j=1}^{\mathcal{S}} \delta_{s_1^*}(j)} \quad \text{, for } i \in \{1, \ldots, \mathcal{S}\}.$$

2. **Update transition probabilities:**

$$a_{ij} = \frac{\sum_{n=1}^{N-1} \mathbf{1}(s_n^* = i \text{ and } s_{n+1}^* = j)}{\sum_{n=1}^{N-1} \mathbf{1}(s_n^* = i)} \quad \text{, for } i, j \in \{1, \ldots, \mathcal{S}\}.$$

3. **Update emission probabilities:**

$$e_{ik} = \frac{\sum_{n=1}^{N} \mathbf{1}(s_n^* = i \text{ and } y_n = k)}{\sum_{n=1}^{N} \mathbf{1}(s_n^* = i)} \quad \text{, for } i \in \{1, \ldots, \mathcal{S}\}, k \in \{1, \ldots, K\}.$$

5.5 Hyperparameter optimization

Hyperparameter optimization was performed through maximization of the BIC. The BIC of the categorical HMM is defined as:

$$\text{BIC} = 2 \log p(Y|\hat{\theta}) - m_{\mathcal{S}} \log(N),$$

where $\hat{\theta}$ is obtained with the EM algorithm described above, $m_{\mathcal{S}}$ is the number of parameters, and N is the number of observations. For each candidate number of hidden states $\mathcal{S}$, ranging from 1 to 30, ten independent model fits were conducted to capture variability in the results. The model yielding the highest BIC score was then selected as the optimal configuration for the HMM.

6 3D pose estimation

6.1 Parameters for 2D pose estimate

The parameters for 2D pose estimation using DeepLabCut are summarized in Table S1.

6.2 Frame loss

To ensure that 2D pose estimates obtained from the five different cameras were properly synchronized despite occasional frame loss (approximately 0.02 % of the frames), frame time-stamping was used to determine the timing of frame loss for a given camera. Pose estimates for these intermittently lost frames were computed using cubic interpolation.

6.3 Parameters for 3D pose estimation

The parameters for triangulating 2D pose estimates and post-processing of the resulting 3D pose are summarized in Table S2.

6.4 Obtaining a gravity-polarized Earth reference frame

The Earth reference frame resulting from Anipose is oriented relative to one of the cameras. To rotate this reference frame so that its z-axis is aligned with the Earth-vertical axis, we computed the principal components of the estimated 3D snout trajectory and used the first two principal components as a proxy for the arena's floor plane (S1A Fig). After confirming that we obtained a right-handed reference frame oriented upward, we also translated this reference frame to obtain trajectories with positive values (minimum altitude and x/y values set to 0; S1B Fig). Visual inspection of the resulting pose estimates was performed to confirm the correct orientation of the reference frame (S1C and S1D Fig).

References

1. Hofmann T, Schölkopf B, and Smola AJ. Kernel methods in machine learning. 2008.
2. Truong C, Oudre L, and Vayatis N. Selective review of offline change point detection methods. Signal Processing 2020;167:107299.
3. Fayat R, Delgado Betancourt V, Goyallon T, et al. Inertial Measurement of Head Tilt in Rodents: Principles and Applications to Vestibular Research. Sensors 2021;21.
4. Menardy F, Varani AP, Combes A, Léna C, and Popa D. Functional alteration of cerebello–cerebral coupling in an experimental mouse model of Parkinson's disease. Cerebral Cortex 2019;29:1752–66.
5. Sra S. A short note on parameter approximation for von Mises-Fisher distributions: and a fast implementation of I s (x). Computational Statistics 2012;27:177–90.
6. Jolliffe IT and Cadima J. Principal component analysis: a review and recent developments. Philosophical transactions of the royal society A: Mathematical, Physical and Engineering Sciences 2016;374:20150202.
7. Pearson K. LIII. On lines and planes of closest fit to systems of points in space. The London, Edinburgh, and Dublin philosophical magazine and journal of science 1901;2:559–72.
8. Hotelling H. Analysis of a complex of statistical variables into principal components. Journal of educational psychology 1933;24:417.
9. Boyd S and Vandenberghe L. Convex optimization. Cambridge university press, 2004.

10. Cattell RB. The scree test for the number of factors. Multivariate behavioral research 1966;1:245–76.
11. Bouveyron C, Latouche P, and Mattei PA. Exact dimensionality selection for Bayesian PCA. Scandinavian Journal of Statistics 2020;47:196–211.
12. Tipping ME and Bishop CM. Probabilistic principal component analysis. Journal of the Royal Statistical Society Series B: Statistical Methodology 1999;61:611–22.
13. Bouveyron C, Latouche P, and Mattei PA. Bayesian variable selection for globally sparse probabilistic PCA. Electronic Journal of Statistics 2018;12:3036–70.
14. Josse J and Husson F. Selecting the number of components in principal component analysis using cross-validation approximations. Computational Statistics & Data Analysis 2012;56:1869–79.
15. Dempster AP, Laird NM, and Rubin DB. Maximum likelihood from incomplete data via the EM algorithm. Journal of the royal statistical society: series B (methodological) 1977;39:1–22.
16. Peel D and McLachlan G. Finite mixture models. John & Sons 2000.
17. MacQueen J et al. Some methods for classification and analysis of multivariate observations. In: *Proceedings of the fifth Berkeley symposium on mathematical statistics and probability*. Vol. 1. 14. Oakland, CA, USA. 1967:281–97.
18. Bishop CM. Pattern recognition and machine learning. Springer, 2006.
19. Bouveyron C, Celeux G, Murphy TB, and Raftery AE. Model-based clustering and classification for data science: with applications in R. Vol. 50. Cambridge University Press, 2019.
20. Frühwirth-Schnatter S, Celeux G, and Robert CP. Handbook of mixture analysis. CRC press, 2019.
