# Supplementary material for: DISSeCT: An unsupervised framework for high-resolution mapping of rodent behavior using inertial sensors
Source: PLoS Biol. 2025 Oct 9;23(10):e3003431. doi: 10.1371/journal.pbio.3003431 (PMC12527166; doi:10.1371/journal.pbio.3003431)
Supplement: S1 Table — (PDF) [file pbio.3003431.s002.pdf]

DeepLabCut	
Version	2.2.0.3
Deep neural network	resnet
Training set	1 950 frames with 12 anatomical landmarks
Data augmentation	Default DLC augmentation
Training iterations	300 000
Learning rate	0.005 for iterations 0–10k then 0.02

S1 Table. Summary of the parameters used for obtaining 2D pose estimates with DeepLabCut.
