# Supplementary material for: DISSeCT: An unsupervised framework for high-resolution mapping of rodent behavior using inertial sensors
Source: PLoS Biol. 2025 Oct 9;23(10):e3003431. doi: 10.1371/journal.pbio.3003431 (PMC12527166; doi:10.1371/journal.pbio.3003431)
Supplement: S2 Table — (PDF) [file pbio.3003431.s003.pdf]

Anipose	
Version	0.4.3
Calibration	5x7 ChArUco board
2D pose filtering	Median filtering with length 13, exclusion for $\Delta > 25$ px from the median or score< 0.25, linear interpolation for excluded samples
Triangulation	RANSAC optimization, scale = 10 for the geometrical constraints, scale = 2 for the temporal smoothing
3D pose filtering	Median filtering with length 19, exclusion for $\Delta > 5\,$cm from the median, linear interpolation for excluded samples

S2 Table. Summary of the parameters used for obtaining 3D pose estimates using Anipose.
