# Supplementary material for: DISSeCT: An unsupervised framework for high-resolution mapping of rodent behavior using inertial sensors
Source: PLoS Biol. 2025 Oct 9;23(10):e3003431. doi: 10.1371/journal.pbio.3003431 (PMC12527166; doi:10.1371/journal.pbio.3003431)
Supplement: S3 Table — (PDF) [file pbio.3003431.s004.pdf]

Keypoint-MoSeq	
Version	0.5.2
Number of keypoints	10 (excluding tail)
Confidence threshold	All coordinate values were set to `nan` for confidence < 0.6
PCA	8 first PCs ($>=$ 90 % of variance explained) kept for model fitting
Kappa (stickiness)	3e5 for the AR step and 3e4 for fitting the full model
Number of iterations	50 for the AR step and 500 for fitting the full model
Memory usage	To keep GPU memory usage under 24 GB, the argument `parallel_message_passing=False` was passed to `kpms.fit_model()`

S3 Table. Summary of the parameters used for running Keypoint-MoSeq on our dataset.
