# Supplementary material for: DISSeCT: An unsupervised framework for high-resolution mapping of rodent behavior using inertial sensors
Source: PLoS Biol. 2025 Oct 9;23(10):e3003431. doi: 10.1371/journal.pbio.3003431 (PMC12527166; doi:10.1371/journal.pbio.3003431)
Supplement: S4 Table — measured on the same workstation used for running DeepLabCut, Anipose, and Keypoint-MoSeq (Dell Precision 5820 Tower with an NVIDIA Quadro RTX 6000 GPU, 24 GB memory). Runtimes shown here were obtained for one run for the rat dataset comprising 8 hours and 38 minutes of inertial measurements (9.3M samples with a sampling rate of 30 Hz). The feature extraction, dimensionality reduction, clustering, and UMAP embedding steps are performed for the 44k segments resulting from the change-point detection step. Results are provided both as the total execution time on the dataset, excluding data loading time, and per hour of recording. (PDF) [file pbio.3003431.s005.pdf]

DISSeCT	
**Step**	**Computation time**
Preprocessing and EKF filtering	17 min 39 s (122 s/h)
Kernel change-point detection	4 min 58 s (34.5 s/h)
Feature extraction	3 min 52 s (26.8 s/h)
Dimensionality reduction and clustering	52 s (6.0 s/h)
UMAP embedding	1 min 27 s (10.1 s/h)
Total	28 min 48 s (200 s/h)

S4 Table. Computation times for the different steps of DISSeCT, measured on the same workstation used for running DeepLabCut, Anipose and Keypoint-MoSeq (Dell Precision 5820 Tower with an NVIDIA Quadro RTX 6000 GPU, 24 GB memory). Runtimes shown here were obtained for one run for the rat dataset comprising 8 hours and 38 minutes of inertial measurements (9.3M samples with a sampling rate of 30 Hz). The feature extraction, dimensionality reduction, clustering and UMAP embedding steps are performed for the 44k segments resulting from the change-point detection step. Results are provided both as the total execution time on the dataset, excluding data loading time, and per hour of recording.
