# Supplementary material for: DISSeCT: An unsupervised framework for high-resolution mapping of rodent behavior using inertial sensors
Source: PLoS Biol. 2025 Oct 9;23(10):e3003431. doi: 10.1371/journal.pbio.3003431 (PMC12527166; doi:10.1371/journal.pbio.3003431)
Supplement: S5 Table — measured on the same workstation used for running DeepLabCut, Anipose, and DISSeCT (Dell Precision 5820 Tower with an NVIDIA Quadro RTX 6000 GPU, 24 GB memory), and excluding data loading/formatting and PCA. Runtimes shown here were obtained for one run for the rat dataset comprising 8 h and 15 min of video recordings (891 052 frames recorded at 30 HZ). The number of iterations correspond to ones recommended by KpMS’s online documention (keypoint-moseq.readthedocs.io). Results are provided both as the total execution time on the dataset, excluding data loading time, and per hour of recording. (PDF) [file pbio.3003431.s006.pdf]

**Keypoint-MoSeq (GPU with CUDA 12)**	
**Step**	**Computation time**
Model fitting: AR-HMM step (50 iterations)	4 min 7 s (0.8 s/h/it)
Model fitting: full model (500 iterations)	2 h 17 min 11 s (3.0 s/h/it)
Total	2 h 21 min 18 s (1027.5 s/h)
**Keypoint-MoSeq (CPU only)**	
**Step**	**Computation time**
Model fitting: AR-HMM step (50 iterations)	10 min 11 s (1.5 s/h/it)
Model fitting: full model (500 iterations)	1 d 2 h 9 min 51 s (22.8 s/h/it)
Total	1 d 2 h 20 min 2 s (11 490.5 s/h)

S5 Table. Computation times for the different steps of Keypoint-MoSeq, measured on the same workstation used for running DeepLabCut, Anipose and DISSeCT (Dell Precision 5820 Tower with an NVIDIA Quadro RTX 6000 GPU, 24 GB memory), and excluding data loading/formatting, and PCA. Runtimes shown here were obtained for one run for the rat dataset comprising 8 h and 15 min of video recordings (891 052 frames recorded at 30 Hz). The number of iterations correspond to ones recommended by KpMS's online documention (keypoint-moseq.readthedocs.io). Results are provided both as the total execution time on the dataset, excluding data loading time, and per hour of recording.
