# Supplementary material for: DISSeCT: An unsupervised framework for high-resolution mapping of rodent behavior using inertial sensors
Source: PLoS Biol. 2025 Oct 9;23(10):e3003431. doi: 10.1371/journal.pbio.3003431 (PMC12527166; doi:10.1371/journal.pbio.3003431)
Supplement: S6 Table — For each cluster, the video snippets corresponding to the top 100 segments with the highest posterior probabilities of belonging to this cluster were examined, given a unique free label and linked with a unique main behavioral category among the following options: orienting, rearing, grooming, locomoting, idle or other (see Fig 1C and Methods). The table shows the first and second most frequent labels per cluster, sorting clusters according to their associated dominant category. For each cluster, the right column shows the percentage of segments belonging to this associated dominant category. The second and third columns from the left indicate the fraction of segments assigned to each cluster (% Segment) and the fraction of the total recording time that they represent (% Duration). (PDF) [file pbio.3003431.s007.pdf]

Cluster	% Segment	% Duration	Human labels (examination of top 100 segments): Most frequent label	%	Second most frequent label	%	
			**Locomotion**				% locomotion
46	3.5	6.1	Locomotion	100	-	-	100
37	3.3	9.8	Locomotion with head down	100	-	-	100
13	3.1	2.0	Locomotion	99	End of rearing	1	99
5	2.9	1.6	Locomotion with downward pitch	42	Rearing end (forepaws touching floor)	25	61
			**Immobility**				% immobile
6	1.6	4.5	Immobility	100	-	-	100
38	1.4	9.3	Immobility	48	Idle with small movements	31	90
20	2.4	3.6	Short bouts of immobility	73	Idle with small movements	13	86
			**Grooming**				% grooming
17	1.2	2.7	Body licking left	100	-	-	100
0	1.0	1.7	Body licking right	99	Licking right forepaw	1	100
43	2.7	4.9	Nose/face wiping with frontpaws	87	Ear scratching with left hindpaw	13	100
26	1.3	1.0	Short bouts of body licking	93	Nose/face wiping with frontpaws	6	99
19	1.0	0.2	Scratching-associated reorientation	48	Body licking-associated reorientation	28	95
30	1.4	1.6	Licking left hindpaw	94	Investigating corner	5	94
45	1.2	2.2	Head/neck scratching with hindpaw	89	Reorientation (head down)	8	89
32	1.2	0.3	Body licking-associated reorientation	65	Nose/face wiping-associated reorientation	22	87
33	1.5	0.3	Body licking-associated reorientation	58	Nose/face wiping-associated reorientation	20	79
3	1.1	0.9	Licking left hindpaw	68	Investigating corner	22	77
8	2.4	1.1	Nose/face wiping with frontpaws	62	Sniffing head down	31	69
29	0.7	0.1	Scratching-associated reorientation	51	Nose/face wiping-associated reorientation	14	68
25	1.5	6.2	Nibbling fur	36	Investigating corner	30	66
21	1.2	0.3	Short bouts of body licking	48	Investigating corner	36	54
			**Rearing**				% rearing
9	2.9	6.3	Rearing	100	-	-	100
14	3.4	2.8	Rearing	100	-	-	100
41	2.3	0.7	Rearing	100	-	-	100
22	2.3	0.8	Rearing	99	Rightward head turn, forepaws on floor	1	99
28	2.9	1.0	Rearing end, Leftward	99	Downward, leftward head rotation, forepaws on floor	1	99
18	3.3	1.1	Rearing end, rightward	95	Downward, rightward head rotation, forepaws on floor	5	95
2	2.3	1.0	Rearing initiation (forepaws leaving floor)	56	Upward head rotation (at least one forepaw not leaving floor)	24	76
10	2.5	1.0	Rearing initiation (forepaws leaving floor)	66	Upward head rotation (at least one forepaw not leaving floor)	29	71
12	3.1	2.9	Rearing initiation (forepaws leaving floor)	49	Upward head rotation (at least one forepaw not leaving floor)	29	71
24	2.7	4.7	Rearing initiation (forepaws leaving floor)	51	Head pitched up	29	65
47	1.5	0.9	Rearing	50	Investigating corner	30	63
15	1.6	0.3	Rearing initiation (forepaws leaving floor)	36	Upward head rotation (at least one forepaw not leaving floor)	33	50
			**Reorientation** (mainly non rearing or grooming-related)				% reorientation
4	3.2	1.7	Upward head rotation	100	-	-	100
1	2.8	0.9	Leftward head turn	99	Leftward head turn during scratching	1	99
40	2.4	2.7	Leftward head turn, head pitched down	84	Rightward head turn, head pitched down, locomoting	15	99
7	2.2	2.3	Rightward head turn, head pitched down	90	Rightward head turn, head pitched down, locomoting	8	98
16	2.7	2.1	Small upward pitch rotation	84	Small upward pitch rotation with locomotion	14	98
23	3.0	0.9	Upward head rotation	97	Rearing	2	97
42	2.4	0.7	Rightward head turn	93	Rearing end, rightward	7	93
27	2.6	0.6	Leftward head turn	84	Rearing end, leftward	14	84
44	2.2	0.7	Rightward head turn	80	Grooming-associated reorientation	20	80
11	2.5	1.6	Leftward, downward head rotation	66	Rearing end, leftward	25	75
34	1.8	0.4	Leftward head turn	62	Grooming-associated reorientation	21	62
39	1.5	0.4	Rightward head turn	61	Grooming-associated reorientation	21	61

S6 Table. Post hoc examination of individual clusters (rat dataset). Caption on next page.

S6 Table. Post hoc examination of individual clusters (rat dataset). For each cluster, the video snippets corresponding to the top 100 segments with the highest posterior probabilities of belonging to this cluster were examined, given a unique free label and linked with a unique main behavioral category among the following options: “orienting”, “rearing”, “grooming”, “locomoting”, “idle” or “other” (see Fig 1C and subsection “Inspection and labeling of video segments” in the Methods section). The table shows the first and second most frequent labels per cluster, sorting clusters according to their associated dominant category. For each cluster, the right column shows the percentage of segments belonging to this associated dominant category. The second and third columns from the left indicate the fraction of segments assigned to each cluster (% Segment) and the fraction of the total recording time that they represent (% Duration).
