# Supplementary figures and images for: DISSeCT: An unsupervised framework for high-resolution mapping of rodent behavior using inertial sensors
Source: PLoS Biol. 2025 Oct 9;23(10):e3003431. doi: 10.1371/journal.pbio.3003431 (PMC12527166; doi:10.1371/journal.pbio.3003431)

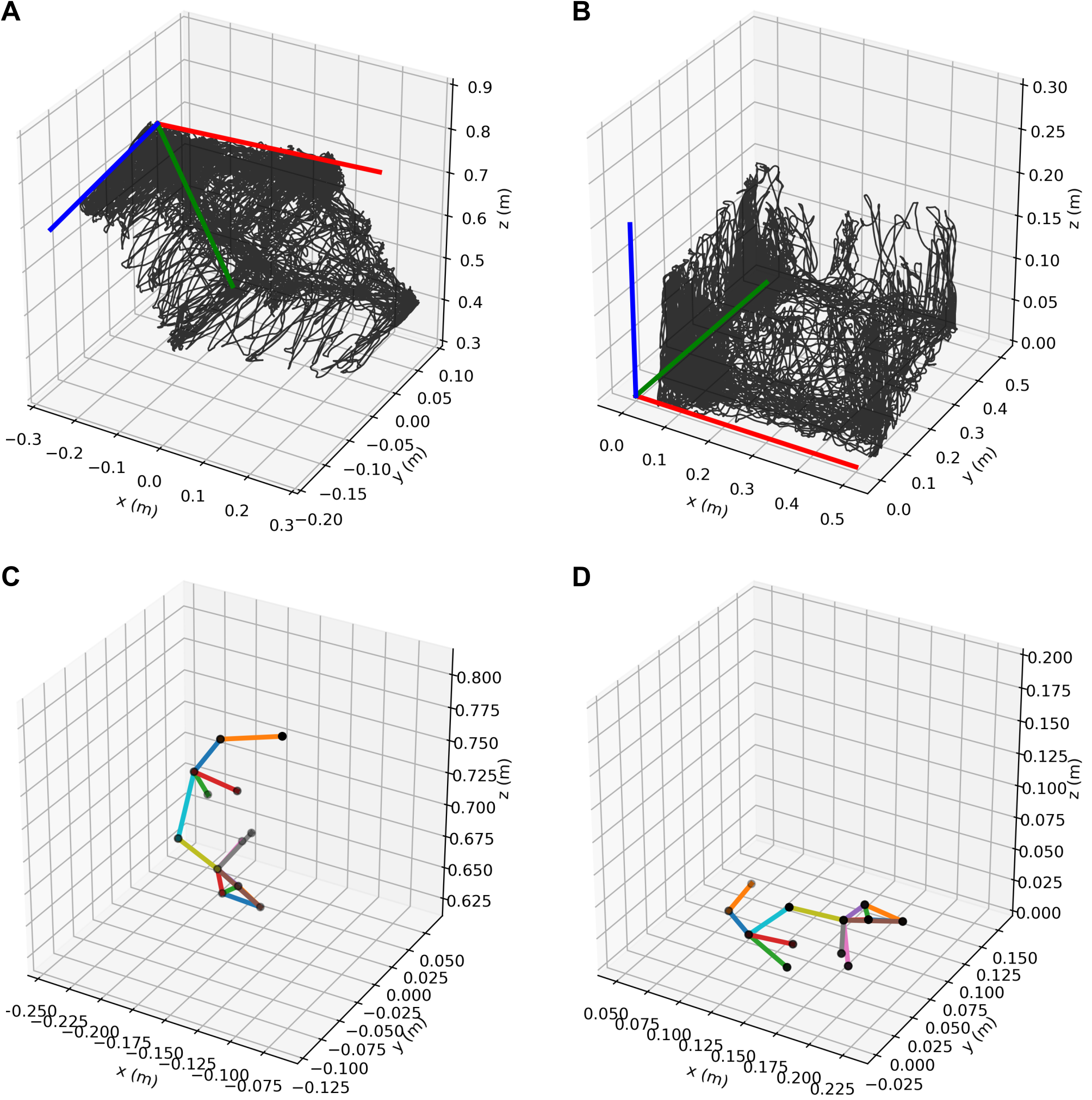

Supplement: S1 Fig — (A) Snout trajectory (black) obtained using Anipose, shown with the orientations of its principal components: PC1 (red), PC2 (green), and PC3 (blue). (B) The same trajectory after transformation into a gravity-aligned reference frame. (C) Example 3D pose estimated using Anipose. (D) The same pose after transformation into the gravity-aligned reference frame. (TIFF) [file pbio.3003431.s010.tiff]

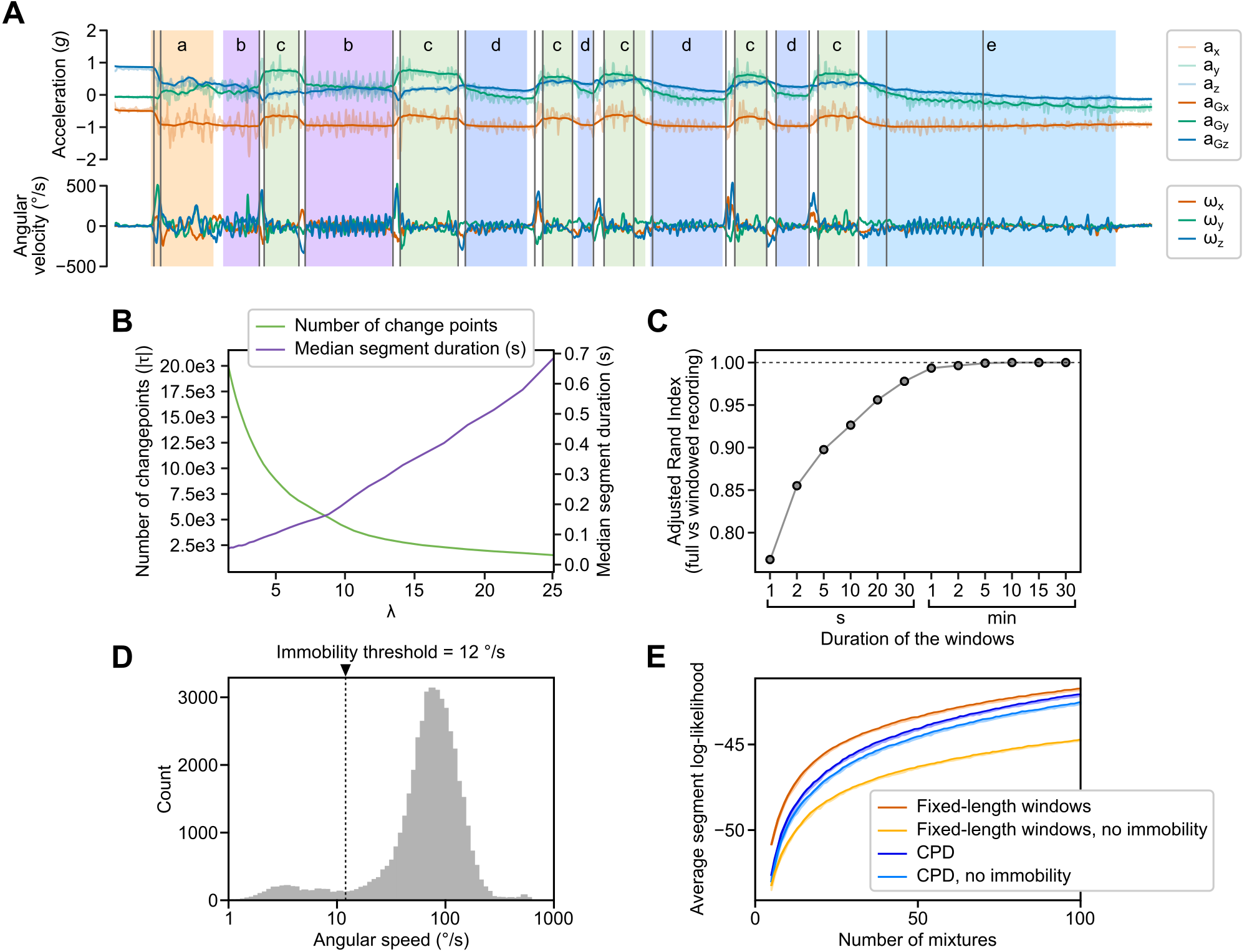

Supplement: S2 Fig — (A) Video-based, frame-level human annotations (colored areas) and detected change points (vertical gray lines) overlaid on IMU data from the 14-second scratching episode shown in S1 Movie. Lowercase letters indicate the following behaviors: (a) sniffing with head pitched downward, (b) left body scratching, (c) left hind paw licking, (d) head scratching on the left side, and (e) decelerating head scratching on the left side. Change points were detected using Gaussian kernel-based Pruned Exact Linear Time (PELT) CPD applied to z-scored angular velocity (ω) and gravitational acceleration (aG) data. Total acceleration (a) is shown alongside aG in lighter shades. (B) Number of detected change points (green) and median segment duration (purple) as a function of the penalty parameter (λ), computed from z-scored aG and ω data in a 30-minute example recording. (C) Adjusted Rand Index (ARI) quantifying the similarity between segmentations obtained by running CPD on the full 30-minute recording versus running CPD in parallel on non-overlapping fixed-length windows. Notably, parallel CPD, which is computationally faster, produces nearly identical segmentations to the full-recording approach when window sizes are at least 5 minutes. (D) Histogram of mean angular speed values (i.e., the norm of gyroscope measurements) computed over non-overlapping, fixed-length windows of 700 ms. The x-axis is logarithmic to highlight the bimodal distribution, which reflects periods of immobility and movement. A threshold of 12 °/s on the standard deviation of angular speed was applied to isolate immobility periods. (E) Average log-likelihood (LL) computed across CPD segments or fixed-length windows from 10 independent GMM fits, plotted as a function of the number of mixture components K. LL values are shown with and without the ablation of immobility segments or windows prior to modeling (see main text for more details). For each K, the shaded region spans the full range of LL values across the [file pbio.3003431.s011.tiff]

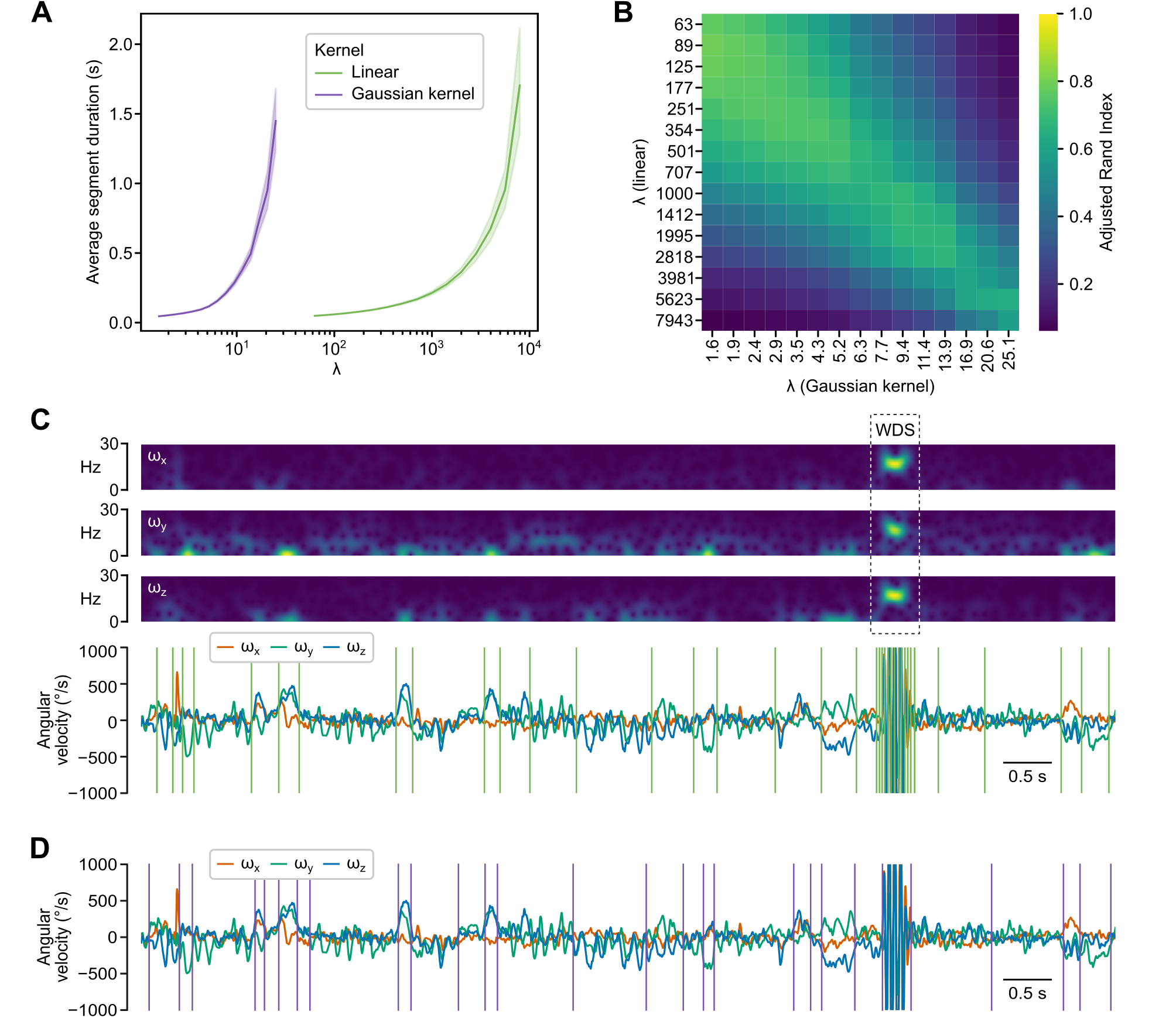

Supplement: S3 Fig — (A) Average segment duration as a function of the penalty parameter (λ) for a 5-minute recording. Results are shown for linear CPD applied to the z-scored time-frequency representation of gyroscope data (green) and for Gaussian kernel CPD directly applied to z-scored gyroscope data (purple). As CPD is deterministic, it was run once for each type of kernel. Shaded areas represent 95% confidence intervals. (B) Adjusted Rand Index comparing the segmentations obtained from the two methods in panel A. (C) Example recording containing a wet dog shake (WDS) event. Top: Time-frequency representation of gyroscope signals, with the WDS’ spectral signature highlighted by a dashed rectangle. Bottom: Corresponding gyroscope signals, with segmentation results from linear CPD on their z-scored time-frequency representation shown as green vertical lines. The time-frequency representation was computed using a Short-Time Fourier Transform (STFT) with a DPSS window (Length = 1 s, Half-bandwidth = 125 ms). Frequencies above 30 Hz were excluded. (D) Same recording as in C, with segmentation results from Gaussian kernel CPD on the z-scored gyroscope data shown as purple vertical lines. (TIFF) [file pbio.3003431.s012.tiff]

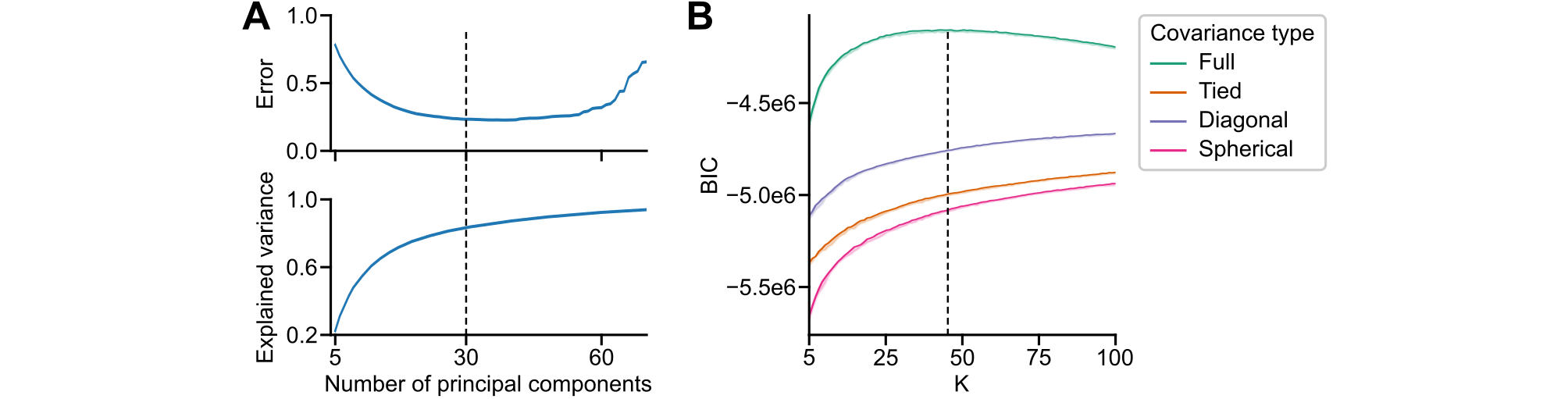

Supplement: S4 Fig — (A) Selection of the number of principal components for dimensionality reduction, based on cross-validation error computed using the procedure described in [107]. The top panel shows cross-validation error as a function of the number of principal components; the bottom panel shows the corresponding fraction of explained variance. (B) Bayesian Information Criterion (BIC) values for increasing numbers of mixture components K and different covariance matrix types (10 independent GMM fits per K value and covariance matrix type). For each K, the shaded region spans the full range of BIC values across the 10 fits. Solid lines connect the maximum BIC values at each K, indicating the best-fitting model. (TIFF) [file pbio.3003431.s013.tiff]

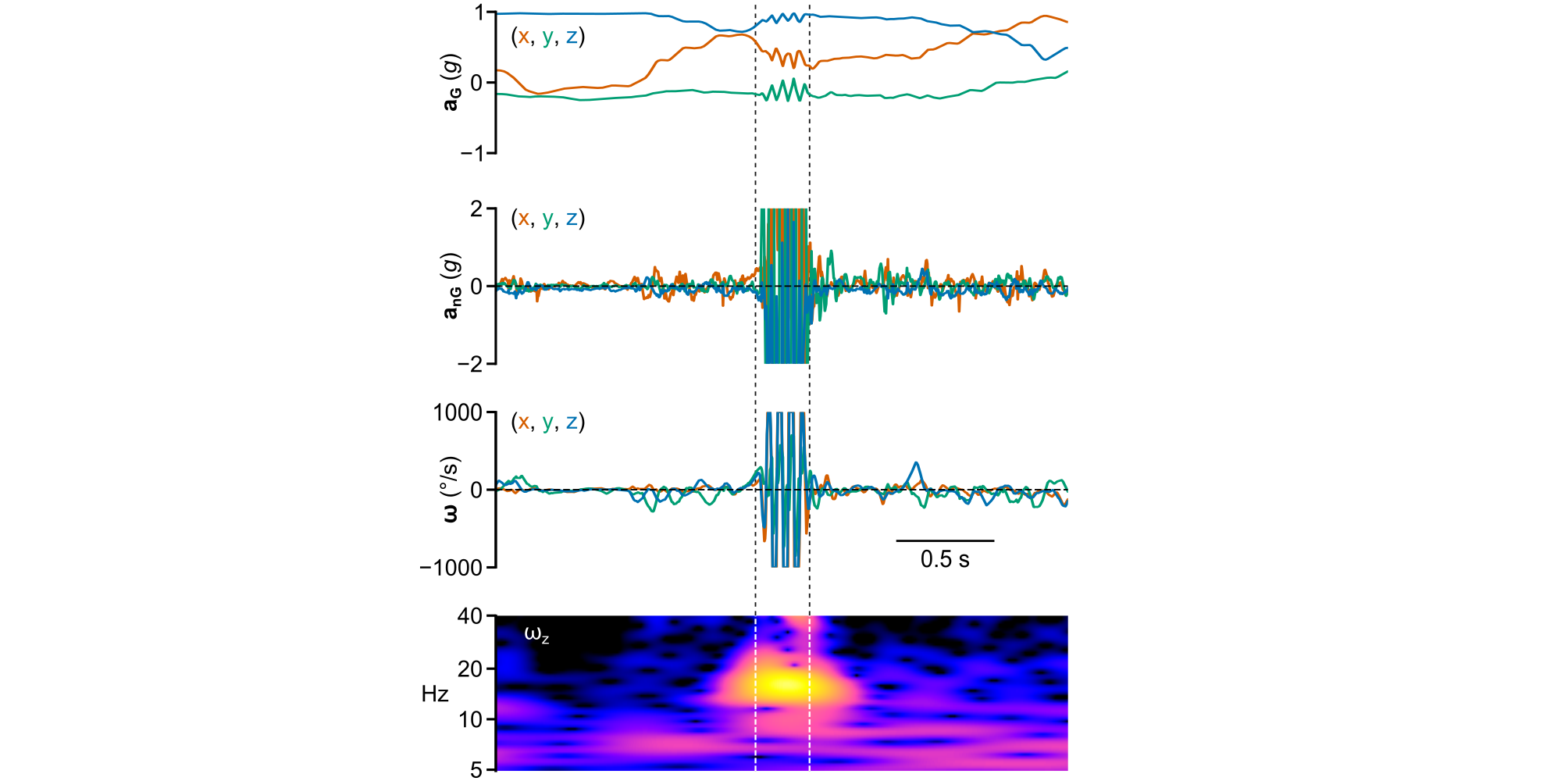

Supplement: S5 Fig — Traces of head gravitational acceleration (aG), non-gravitational acceleration (anG), and angular velocity (ω) are shown. The bottom panel displays the log-magnitude of continuous wavelet transform coefficients computed on the yaw component of angular velocity. Vertical lines mark the start and end of the segment identified using Gaussian kernel-based PELT change-point detection (see S3 Fig). (TIFF) [file pbio.3003431.s014.tiff]

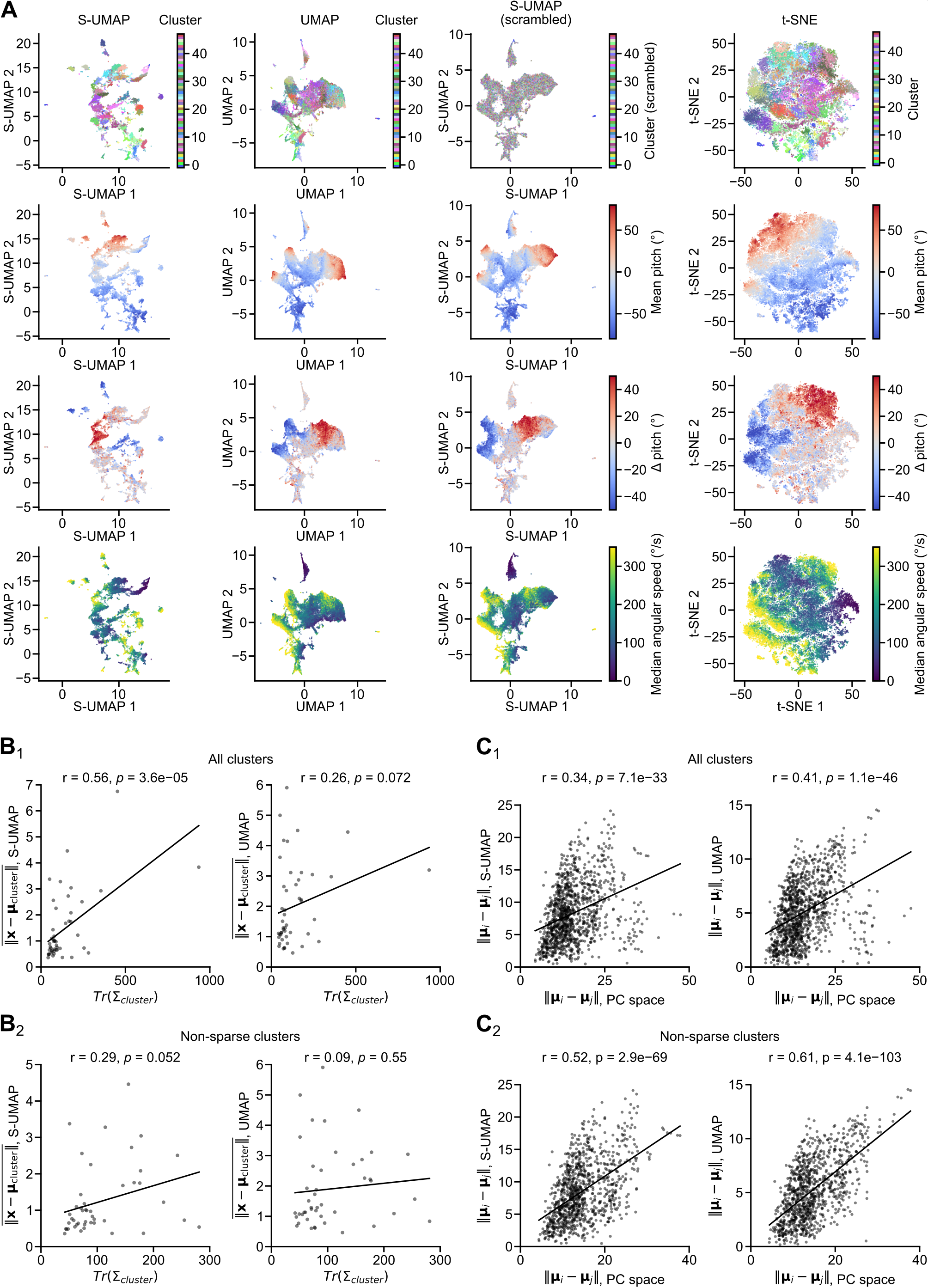

Supplement: S6 Fig — (A) Columns (left to right): (1) Supervised UMAP (S-UMAP), incorporating both feature values and cluster identities; (2) Unsupervised UMAP using only feature values; (3) S-UMAP with shuffled cluster identities; and (4) t-SNE projection based on feature values (early_exaggeration = 12, perplexity = 30). Top row: segments colored by assigned cluster identity. Subsequent rows: segments colored by selected example feature values. (B) Cluster spread in the PC space (x-axis; trace of each cluster’s covariance matrix) compared with spread in UMAP and S-UMAP embeddings (y-axis; average Euclidean distance of cluster members from their centroid), including either all clusters (B1) or only non-sparse clusters (B2), as defined in Fig 1C. (C) Pairwise inter-cluster distances in the PC space (x-axis; distances between centroids μi and μj for clusters i and j, with i>j) compared with those in UMAP and S-UMAP embeddings (y-axis), including either all clusters (C1) or only non-sparse clusters (C2), as defined in Fig 1C. (TIFF) [file pbio.3003431.s015.tiff]

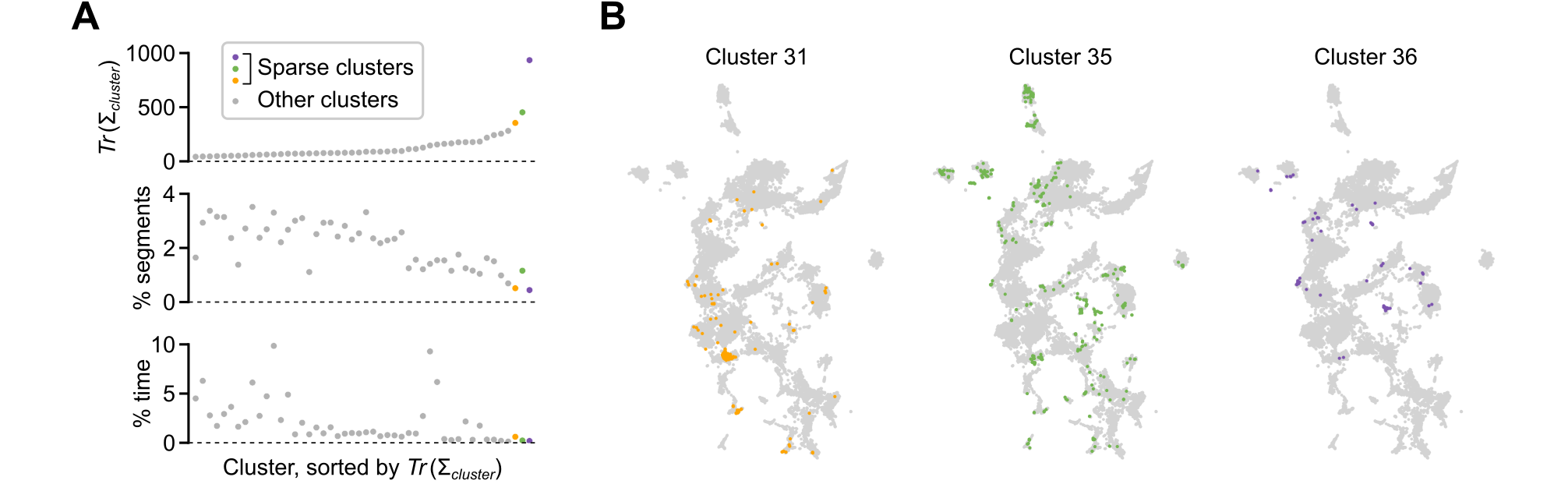

Supplement: S7 Fig — (A) Top: trace of the covariance matrix for each cluster, indicating cluster dispersion. The three highlighted clusters (in orange, green, and purple) correspond to the most dispersed clusters. Middle: percentage of the total number of segments contained in each cluster. Bottom: percentage of the total recording duration associated to each cluster. Clusters are sorted by ascending trace values in all panels. (B) Supervised UMAP embeddings displaying segments belonging to the three most dispersed clusters (in color) overlaid onto other segments (in gray). (TIFF) [file pbio.3003431.s016.tiff]

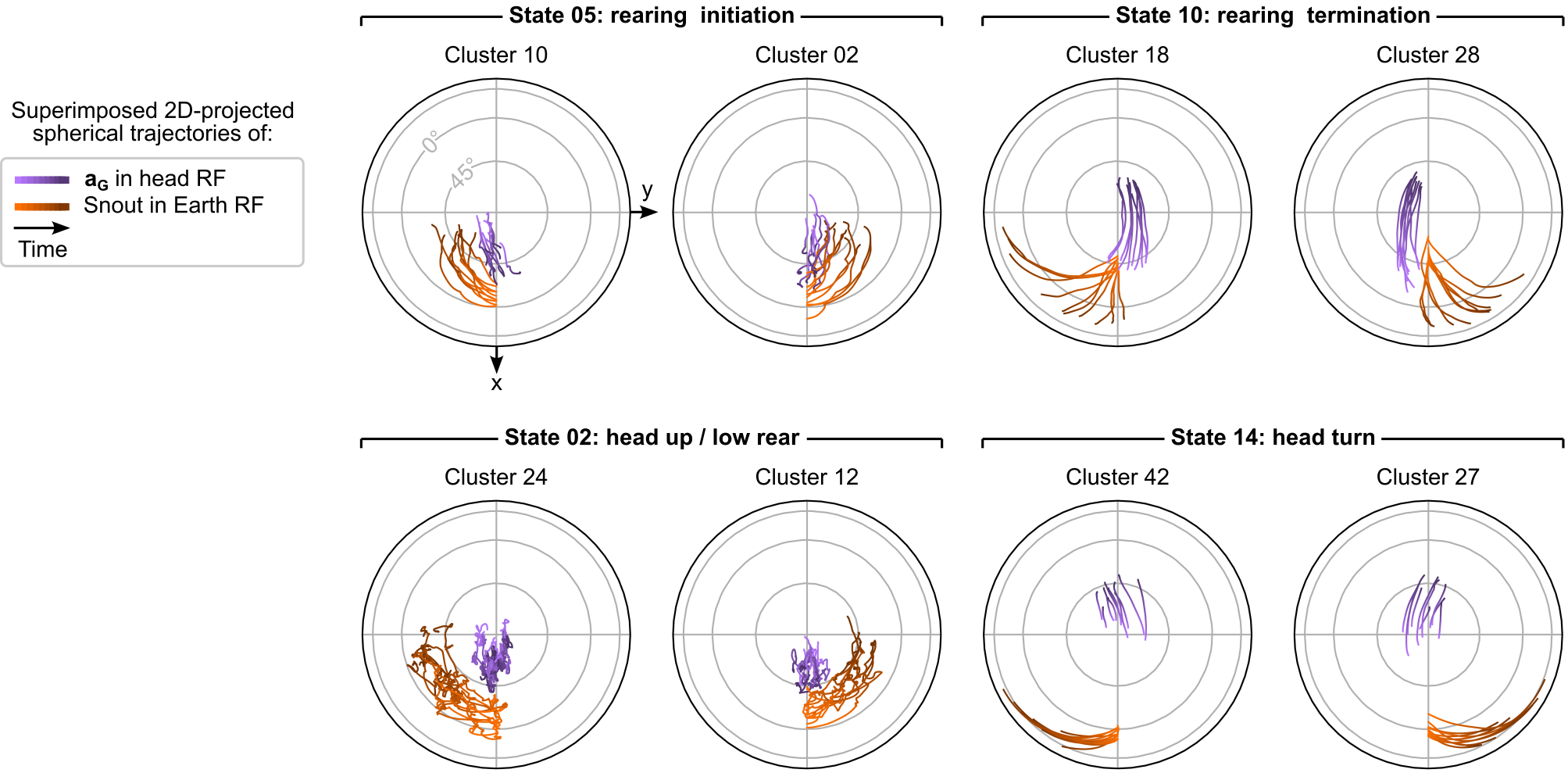

Supplement: S8 Fig — Plots represent the joint visualization of the trajectories of aG in the head reference frame (purple) and of the animal’s heading in the Earth reference frame (orange) for the top 10 segments of pairs of clusters associated with four hidden states (see subsection 2.2 in S1 Text; RF: reference frame). Each pair represents the same head movement executed toward the left or right. (TIFF) [file pbio.3003431.s017.tiff]

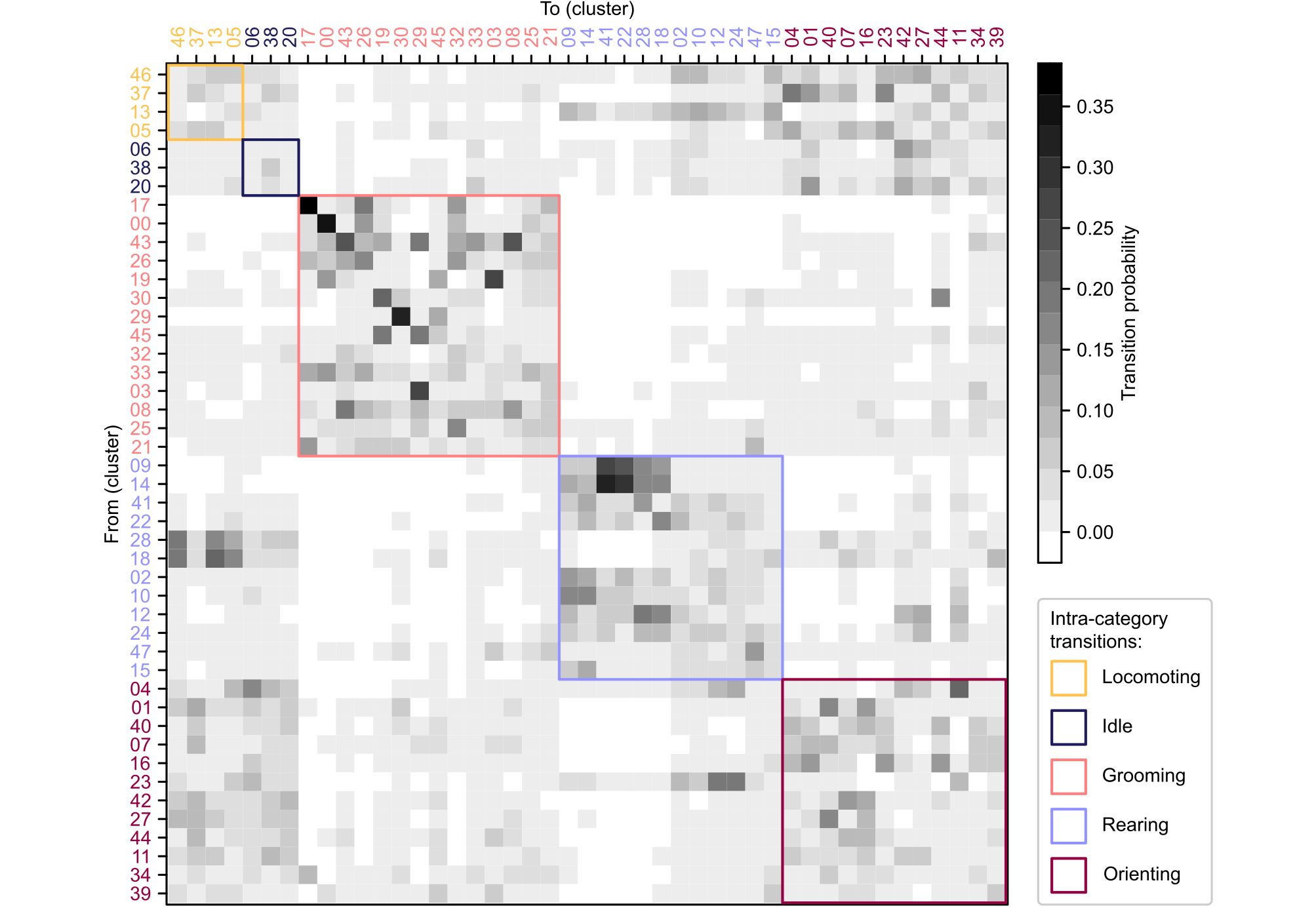

Supplement: S9 Fig — This graph represents the transition probability between clusters. Cluster numbers are ordered and color-coded according to their dominant main behavioral category (Fig 1C). Colored squares highlight intra-category transitions. (TIFF) [file pbio.3003431.s018.tiff]

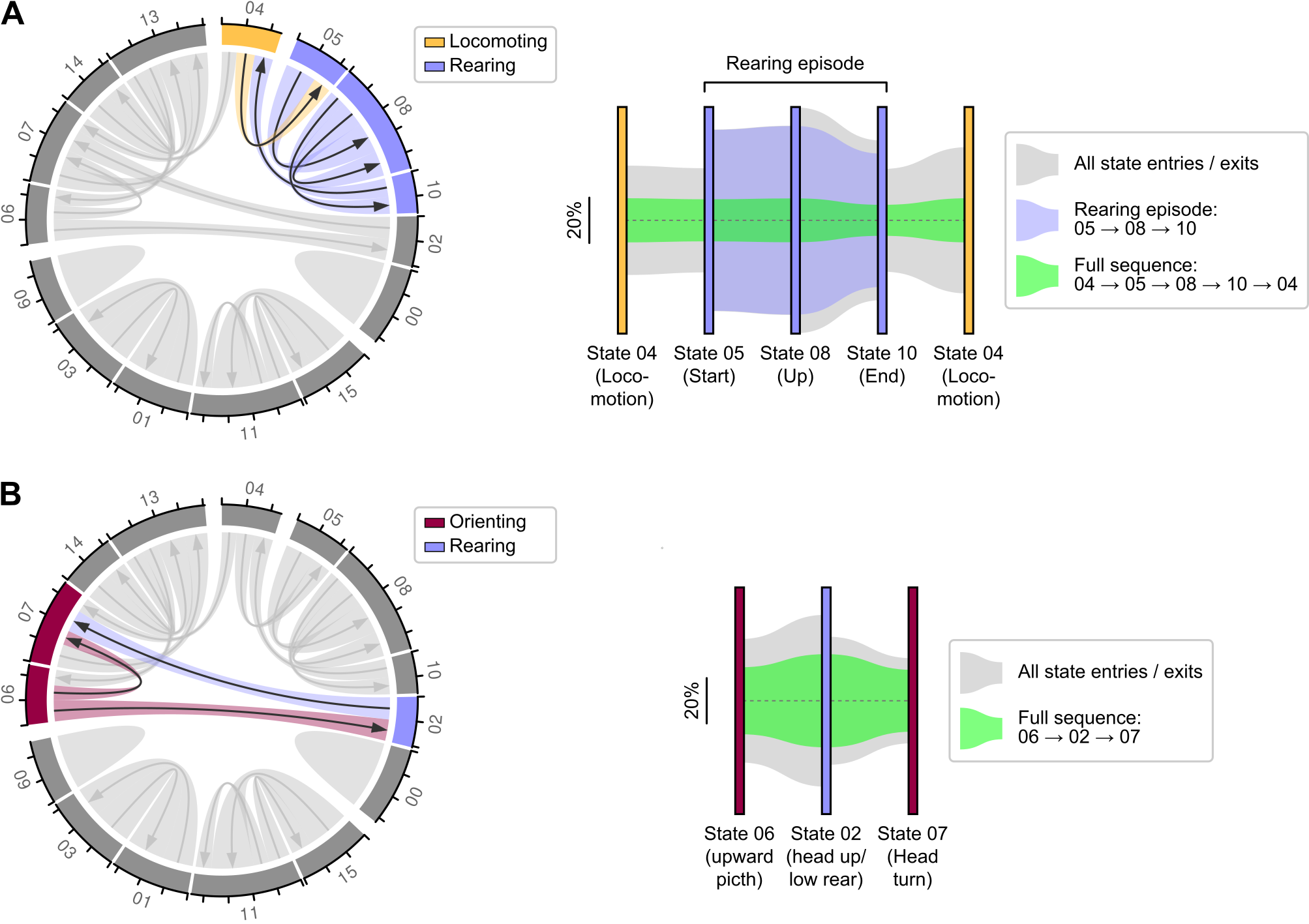

Supplement: S10 Fig — (A) Left: chord plot highlighting state transitions corresponding to alternating rearing episodes and locomotor bouts. Right: Sankey diagram showing the proportion of state transitions occurring within isolated rearing episodes (blue) or within a full locomotion-rearing-locomotion sequence (green). The diagram is read from left to right. Colored vertical bars represent states, while shaded areas connecting states represent the proportion of state exits (right side of bars) and entries (left side of bars). (B) Left: chord plot highlighting state transitions corresponding to sequences during which the animal adopts a pitched-up head posture while keeping its front paws on the ground or close to it (low rear). Right: Sankey diagram showing the proportion of state transitions occurring in the context of this type of sequence (green). (TIFF) [file pbio.3003431.s019.tiff]

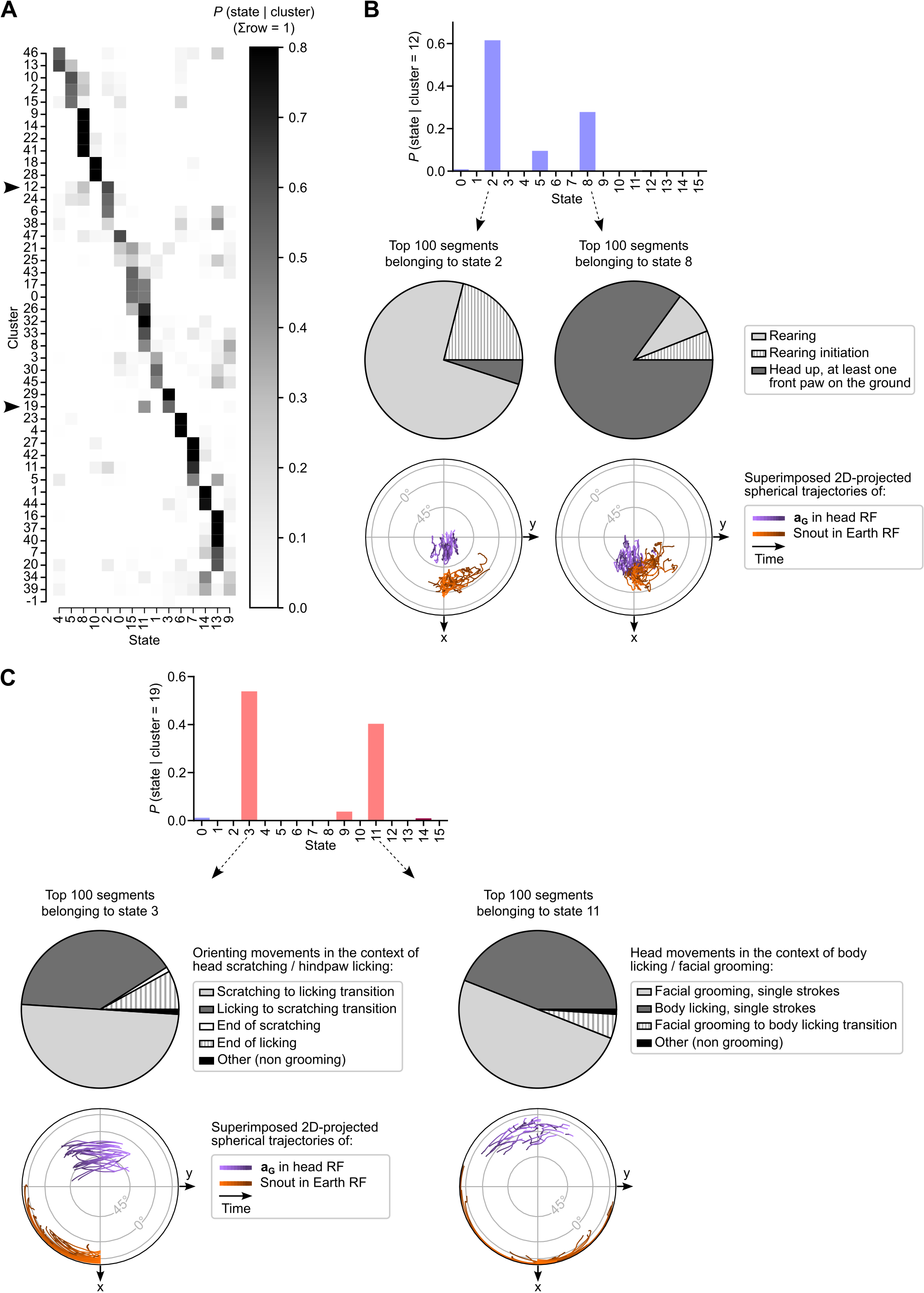

Supplement: S11 Fig — (A) Posterior probability matrix, displaying the likelihood of each state given each cluster, denoted as P(state|cluster). Arrowheads highlight two example clusters (12 and 19) that are associated with multiple states. (B) Top: Bar plot illustrating the posterior probabilities for cluster 12, showing a strong association with states 2 and 8. Middle: Pie charts displaying the proportion of unique labels assigned to the top 100 segments with the highest P(state=2|cluster=12) (left) or P(state=8|cluster=12) (right). Note that while the same labels appear in both groups, their proportions differ drastically. Bottom: joint visualization of the trajectories of aG in the head reference frame (purple) and of the animal’s heading in the Earth reference frame (orange) for the top 30 segments in each group. (C) Top: Bar plot showing the posterior probabilities for cluster 19, indicating a predominant association with states 3 and 11. Middle: Pie charts displaying the proportion of unique labels given to the top 100 segments with the highest P(state=3|cluster=19) (left) or P(state=11|cluster=19) (right). Note that labels differ between the two groups, although the same pattern fills are used. Bottom: Joint visualization of the trajectories of aG in the head reference frame (purple) and of the animal’s heading in the Earth reference frame (orange) for the top 30 segments in each group. (TIFF) [file pbio.3003431.s020.tiff]

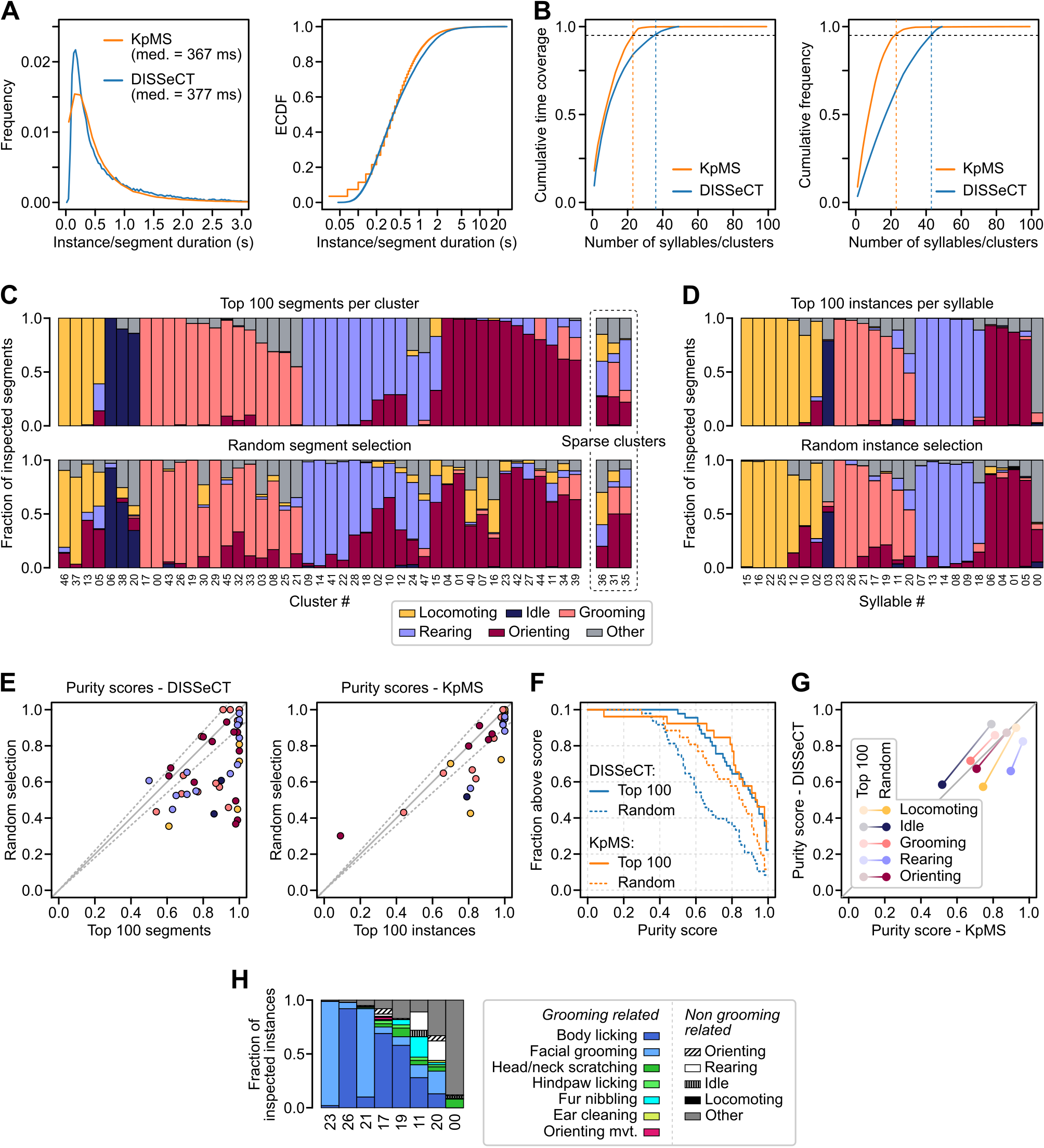

Supplement: S12 Fig — (A) Distribution of segment durations for DISSeCT and syllable instance durations for KpMS, shown as frequency histograms (left) and empirical cumulative distribution functions (ECDFs; right). Median durations are indicated above the histograms. (B) Left: cumulative time coverage—i.e., the cumulative proportion of total recording time—as a function of the number of clusters (DISSeCT) or syllables (KpMS), sorted by decreasing contribution. A minimum of 23 KpMS syllables (vertical orange dashed line) and 36 DISSeCT clusters (vertical blue dashed line) were required to account for at least 95% of the total recording time (horizontal black dashed line). Right: cumulative frequency—i.e., cumulative proportion of total instances or segments—as a function of the number of syllables or clusters, sorted by decreasing contribution. At least 23 KpMS syllables and 43 DISSeCT clusters were needed to account for 95% of all instances and segments, respectively. (C) Stacked bar plots showing the fraction of segments in each DISSeCT cluster assigned to different behavioral categories, based on manual inspection of either the top 100 segments per cluster (top; same data as in Fig 1C) or a random sample (bottom; see Methods). (D) Same as C, but for KpMS syllables. Top: top 100 instances per syllable. Bottom: randomly selected instances (see Methods). (E) Purity scores for each DISSeCT cluster (left; excluding sparse clusters) and KpMS syllable (right), based on either random samples (x-axis) or the top 100 segments/instances (y-axis). Each dot represents a cluster or syllable, colored by its dominant behavioral category (as in C-D). The solid diagonal line indicates identity; dashed lines denote ±10% deviation. (F) Fraction of clusters or syllables with a purity score above a given threshold, plotted as a function of that threshold. Solid lines: top 100 segments/instances. Dashed lines: random samples. (G) DISSeCT vs. KpMS purity scores at the behavioral category level. Each behaviora [file pbio.3003431.s021.tiff]

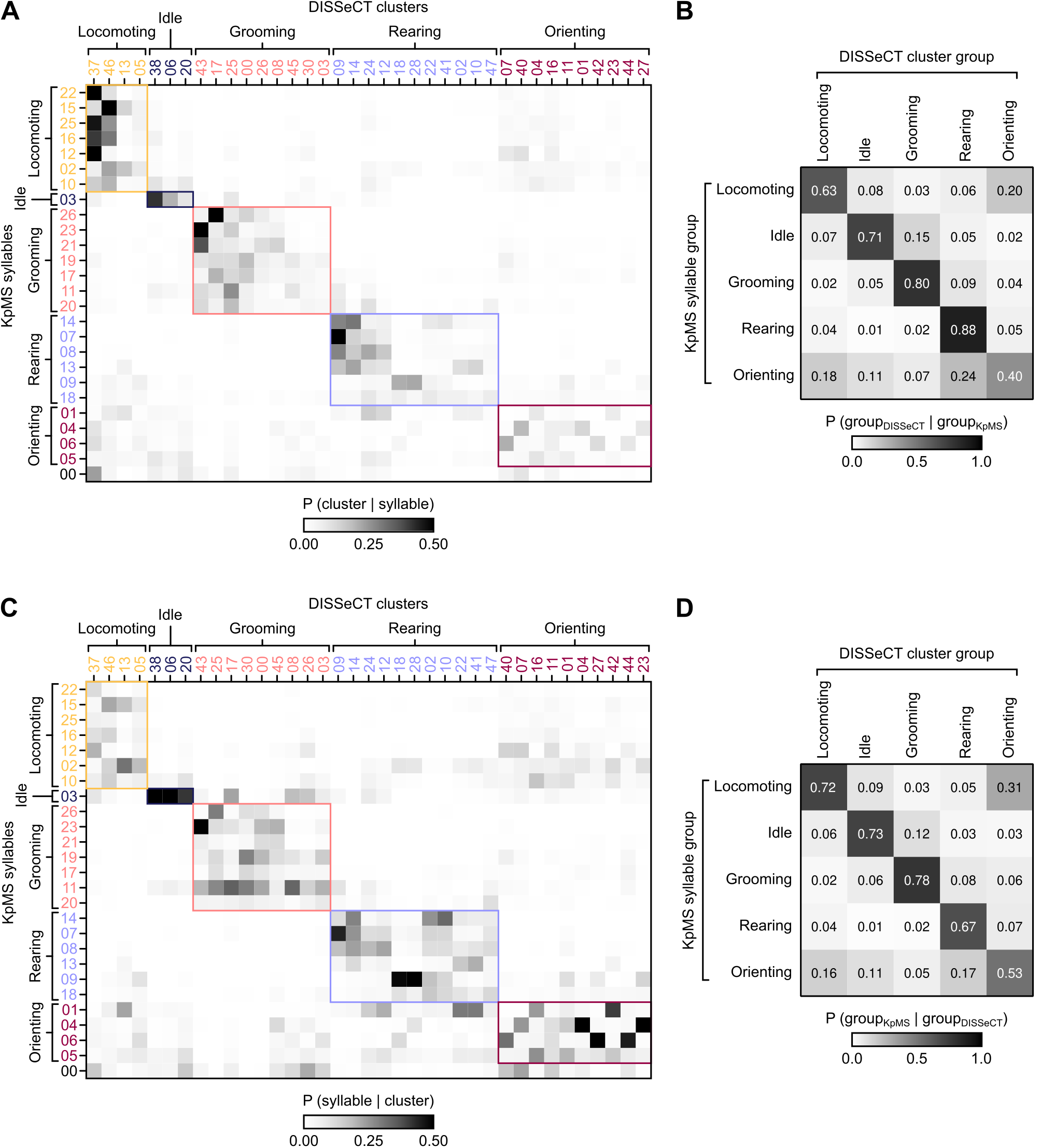

Supplement: S13 Fig — For this analysis, KpMS syllables and DISSeCT clusters accounting for less than 5% of the total recording duration were excluded. DISSeCT outlier segments and sparse clusters were also excluded. (A) Row-normalized contingency matrix showing the conditional probability of DISSeCT clusters given KpMS syllables. Both syllables and clusters are grouped by behavioral category. (B) Same analysis at the behavioral category level. (C) Column-normalized contingency matrix showing the conditional probability of KpMS syllables given DISSeCT clusters. (D) Same analysis at the behavioral category level. (TIFF) [file pbio.3003431.s022.tiff]

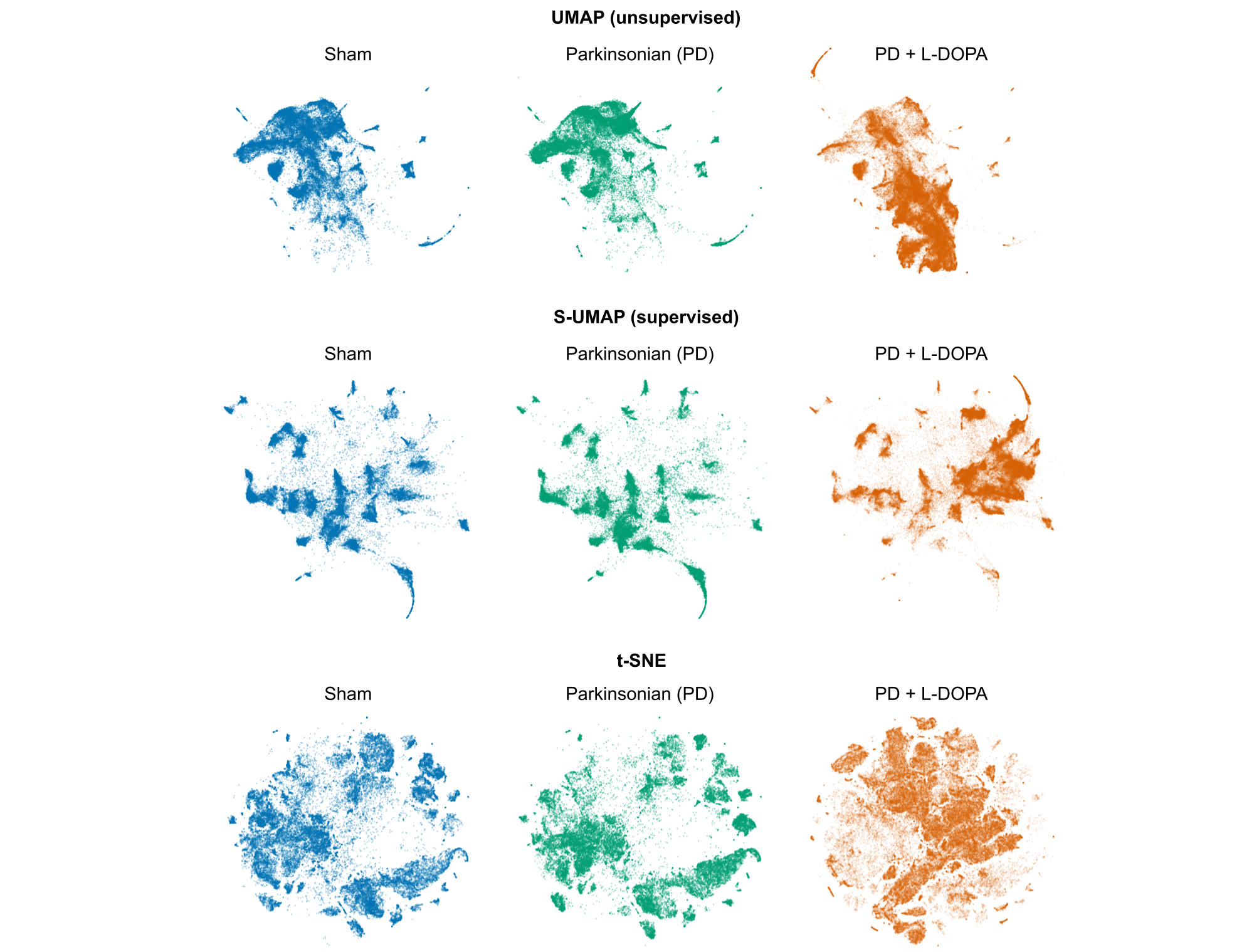

Supplement: S14 Fig — This figure shows the results of two fully unsupervised embedding methods (UMAP: top; t-SNE: bottom) and one semi-supervised method (S-UMAP: middle), applied to Sham (left, blue) and PD mice before (middle, green) and during (right, orange) L-DOPA treatment. For S-UMAP, cluster identities were derived from a Gaussian Mixture Model (GMM) fitted on data from sham mice. Hyperparameters were set as follows: n_neighbors=8, n_components=2 and min_dist=1×10^−3^ for both UMAP and S-UMAP, as well as target_weight=1×10^−9^ for S-UMAP; early_exaggeration=12 and perplexity=30 for t-SNE. (TIFF) [file pbio.3003431.s023.tiff]

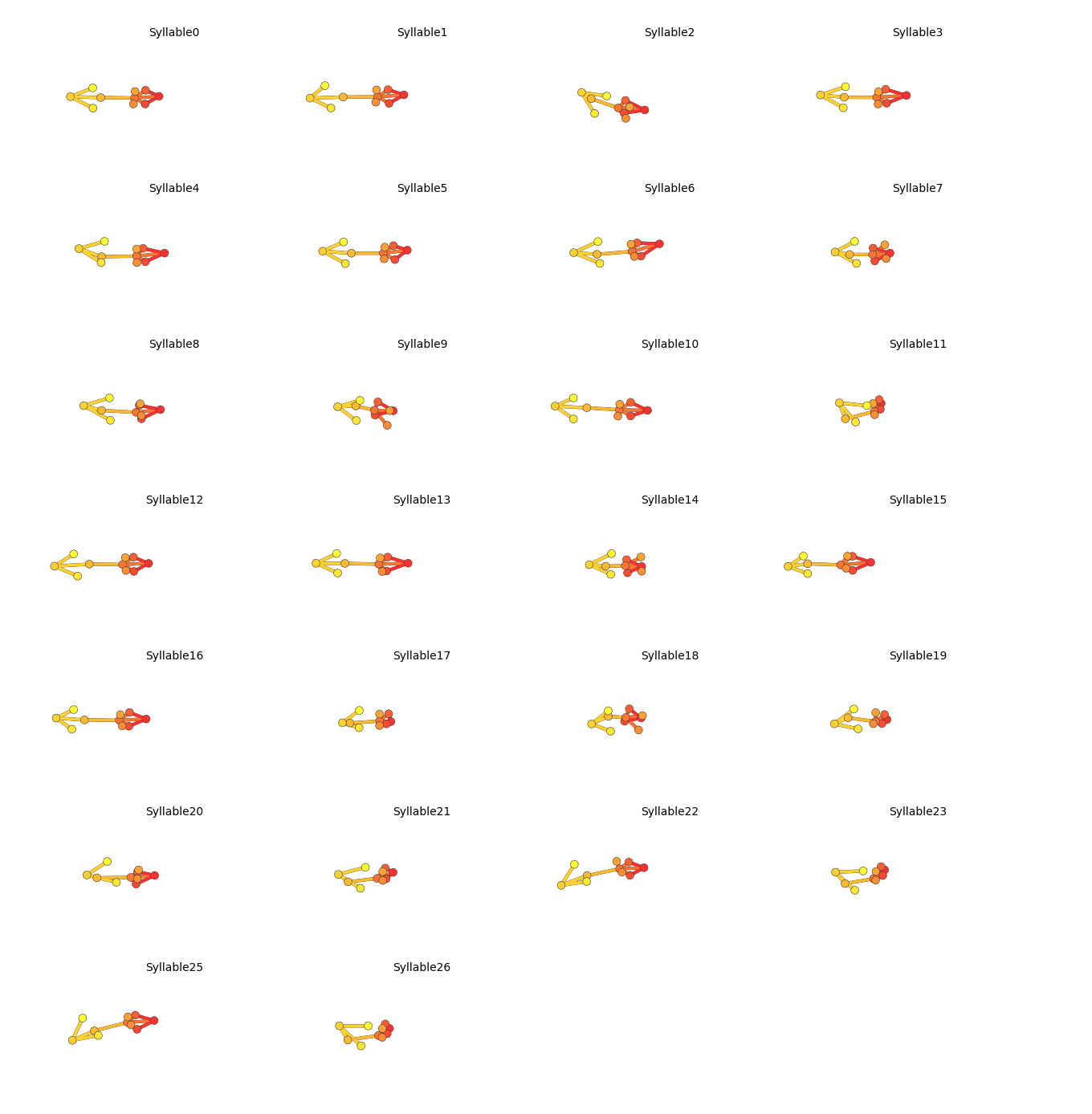

Supplement: S4 Movie — (GIF) [file pbio.3003431.s027.gif]

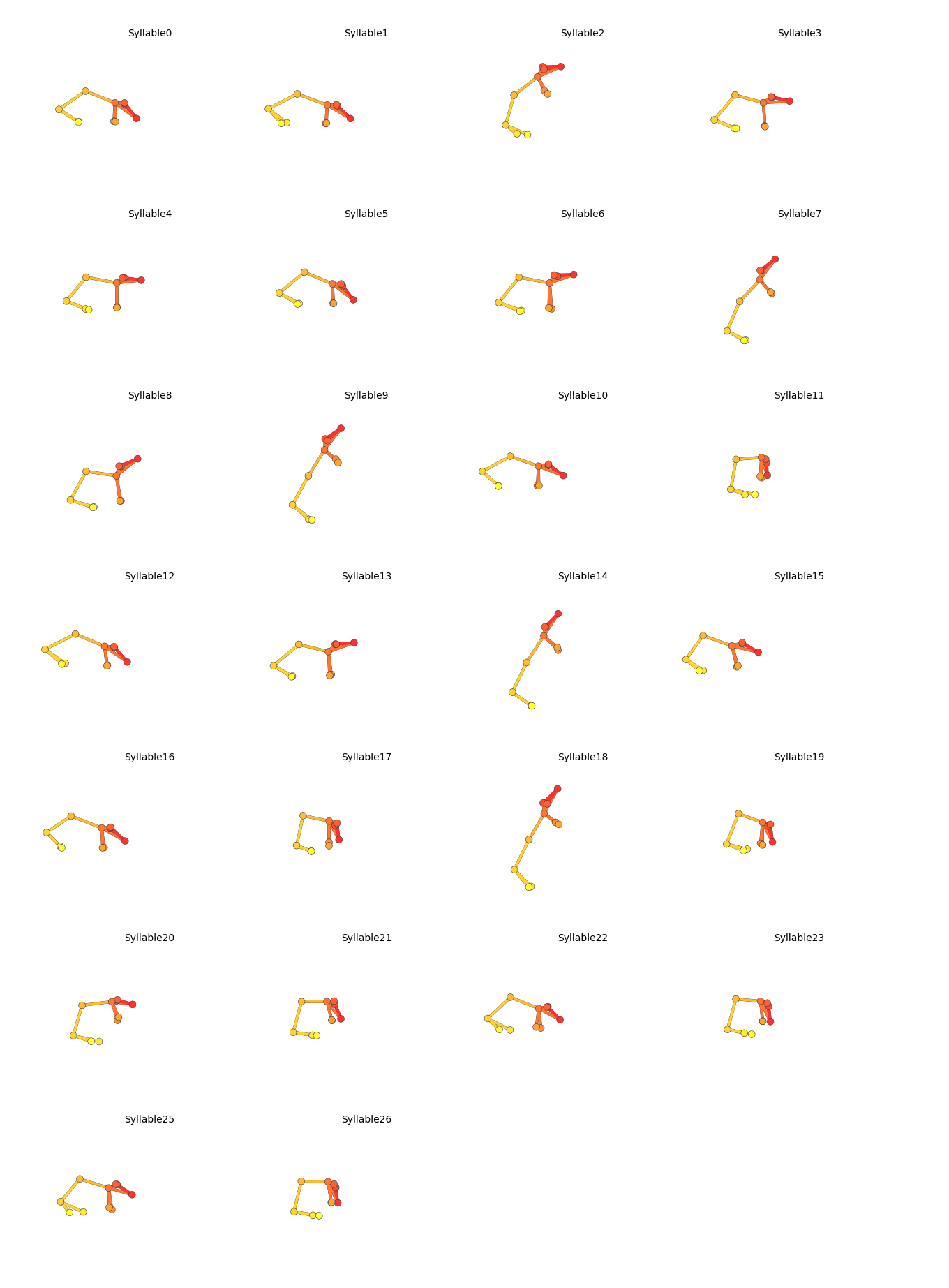

Supplement: S5 Movie — (GIF) [file pbio.3003431.s028.gif]
