# Supplementary material for: DISSeCT: An unsupervised framework for high-resolution mapping of rodent behavior using inertial sensors
Source: PLoS Biol. 2025 Oct 9;23(10):e3003431. doi: 10.1371/journal.pbio.3003431 (PMC12527166; doi:10.1371/journal.pbio.3003431)
Supplement: Attachment — Submitted filename: Rebuttal_Fayat_et_al_PLOS_Biology.pdf [file pbio.3003431.s029.pdf]

Fine decomposition of rodent behavior via unsupervised segmentation and clustering of inertial signals

PBIOLOGY-D-24-03343R1

Romain Fayat[1], Marie Sarraudy[1], Clément Léna[1], Daniela Popa[1], Pierre Latouche[2,3] and Guillaume P. Dugué[1*]

[1]Neurophysiology of Brain Circuits, Institut de Biologie de l'École Normale Supérieure (IBENS), École Normale Supérieure, CNRS, INSERM, Université PSL, 75005 Paris, France
[2]Université Clermont Auvergne, CNRS, Laboratoire LMBP, UMR 6620, 63178X Aubière, France
[3]Institut Universitaire de France (IUF)

Response to Reviewers

We thank the reviewers for their thoughtful and constructive feedback. Below, we address each comment in detail. Reviewer comments are shown in *italic blue*, and our responses follow each comment.

Reviewer 1

The paper effectively describes the use of an unsupervised learning pipeline to analyse rodent behavior from IMU data. The authors present compelling arguments for the usefulness of this approach as compared to multi-angle, video-based pose estimation techniques. Their pipeline appears both sensible and robust, and the accompanying data and GitHub repository are well-formatted and easy to follow, making this method readily adoptable by other laboratories.

From a computational standpoint, while none of the unsupervised techniques employed are individually novel, the overall demonstration of their applicability to IMU data is indeed novel, valuable, and likely poised for widespread adoption.

We do raise one concern regarding the generalizability of their model, along with a few suggestions for additional statistical tests that would further strengthen the applicability of their technique. However, we want to emphasise that the work is already suitable for acceptance as is. These suggestions are meant as optional considerations that the authors may wish to discuss or incorporate at their/editorial discretion.

*Corresponding author: guillaume.dugue@cnrs.fr

0. One discussion point is about how generalizable the learned parameter space is. It would be convincing to run the original model on a new ethological dataset (e.g. mice recorded in a distinct complex enriched environment) to perform a comparison of the original model and the new model trained solely on the novel dataset. They could compare the model likelihoods using an F-test, Wilks test, or a Bayesian information criterion measure.

First of all, we would like to emphasize that **model generalizability is inherently dependent on sensor placement**. Only data acquired from similarly positioned and oriented IMUs can be meaningfully compared. This constraint is not unique to our approach and equally applies to pose-based models, where valid comparisons require animals to share similar size, strain, and anatomical proportions.

The **probabilistic nature of GMMs** makes it straightforward to assess how well a learned model generalizes to new data—a key motivation for incorporating GMMs into our pipeline. Generalizability can be evaluated through two complementary approaches: (1) **cross-likelihood analysis**, which is directly inspired by likelihood ratio tests and assesses how well a GMM trained on one dataset explains another (i.e., the likelihood of the new data under the original model); and (2) **direct structural comparison** between GMMs fitted independently to each dataset, using entropy-based metrics such as the Jensen-Shannon divergence.

Because we use a GMM, and not a single-Gaussian model for continuous data, classical statistical tests for normality—or tests that assume normality—are not directly applicable. In our manuscript, **we illustrate the cross-likelihood approach** by testing how a GMM trained on sham animals generalizes to Parkinsonian mice, both under L-DOPA and untreated. Since the Bayesian Information Criterion (BIC) combines the log-likelihood with a model complexity penalty, and we applied the same GMM across datasets, the penalty term remained constant. As a result, comparing BIC values effectively reduced to comparing log-likelihoods.

This same methodology could be applied to the scenario suggested by the reviewer, by computing the log-likelihood of IMU data collected in a large, enriched environment (which would likely elicit ethologically relevant patterns such as galloping or bounding) under a GMM trained on our more constrained setting (rats in a 70×70 cm empty arena). While this would indeed highlight the expansion of the behavioral repertoire under naturalistic conditions, it would not represent a methodological innovation within the scope of our study, as it employs the same inference framework we have already implemented and validated.

We have expanded the discussion to address these points (p. 14–15, lines 440–447 and p. 17, lines 540–546).

1. A second comment is about demonstrating more convincingly the usefulness of their change-point detection method. It would be sufficient to show that this creates statistically better features for their clustering method than a random segmentation of the dataset into arbitrary time windows, so the authors would just need to retrain their PCA/GMM model on these random segments, and perform statistical tests to show the likelihood is statistically significantly worse on the random features.

From a mathematical standpoint, kernel-based change-point detection (CPD) provides a **globally optimal segmentation of time series**. This theoretical guarantee was a key motivation for integrating CPD into our pipeline. Nonetheless, we acknowledge the reviewer's suggestion that further empirical support for its practical utility would strengthen the manuscript.

Rather than comparing CPD to random segmentation, as suggested, we opted to bench-

mark it against segmentation into **fixed-length windows**, a common default starting point in many behavioral analysis pipelines. We applied the same downstream procedures (feature extraction, PCA, and GMM clustering) to both segmentation strategies and compared the resulting average log-likelihoods (LL) under the respective GMMs.

Initially, fixed-length windows yielded a higher LL than CPD-derived segments. We hypothesized that this was due to the over-representation of immobility segments in the fixed-length approach, which are comparatively easier for the GMM to model. To test this, we excluded immobility windows or CPD segments prior to modeling. Under these conditions, **the CPD-based approach outperformed fixed-length segmentation in terms of LL**, supporting its effectiveness in providing a better ground for the clustering step. **This new analysis is described in a new Methods paragraphs (p. 23–24, lines 733–745). Its results are presented in a new Results paragraph (p. 5, lines 130–142) and a new supplementary figure panel (Fig. S2E)**.

2. Finally, to be more convinced of the Parkinson's study, they could once again train a model on the mice after L-DOPA administration, and compare this model likelihood with the sham model, to show a significant difference in likelihoods from the two models on the Parkinson's model animals, thus demonstrating statistically a difference in behaviours between the sham and diseased animals.

In our manuscript, 6-OHDA-lesioned mice were compared to the sham group using a cross-likelihood approach: a GMM was first fitted to sham data to obtain a "sham model", and the average log-likelihood of lesioned mice data—both before and after L-DOPA administration—was then evaluated under this model (referred to as the Score Under Sham Model, or SUSM; see Fig. 7 and p. 14–15, lines 439–458). **This method quantifies how well the kinematic patterns of lesioned animals is explained by the sham model**. As the reviewer rightly pointed out, this analysis could be performed in the reverse direction—i.e., evaluating how well sham data can be explained by a GMM trained on L-DOPA–treated lesioned animals. As discussed above, such reciprocal comparisons could be further supplemented with structural analyses of the GMMs using entropy-based metrics. However, **given the pronounced divergence in behavioral profiles observed in lesioned animals after L-DOPA treatment—already well captured by the SUSM—we believe that additional metrics would provide limited added value**.

3. As a more general comment, I don't find it that clear in the discussion whether the authors are encouraging the use of their pipeline, or their actual learned model parameters. The latter case will take more convincing of its broader applicability, while the former makes a lot of sense as a clustering pipeline, but would lose the detailed annotations of the clusters they found for their particular dataset, as clusters from a new model can't necessarily be identified with the clusters found in their model. More clarity on this point would be beneficial.

We do encourage the use of our pipeline rather than relying solely on the learned GMM parameters, which are likely influenced by our specific recording conditions and may not fully capture the behavioral repertoire of Long Evans rats. Instead, we recommend running the pipeline from scratch and, ideally, maintaining a minimal parallel video recording to aid in interpreting cluster content. In future studies, post hoc cluster analysis will be facilitated by:

- our demonstration that key behavioral motifs (e.g., locomotion, grooming, sniffing, extended

rearing) exhibit **distinct and interpretable features**, such as characteristic spectral signatures or head tilt patterns.

- our companion software, INSpECT, which streamlines visualization and manual labeling.

As this discussion relates to model generalizability, we also address these considerations on p. 14–15, lines 440–459.

4. *Could they validate the use of their kernel vs other kernels in their change-point detection cost function?*

We did compare our Gaussian kernel to a linear kernel for change-point detection (CPD), as shown in Fig. S3 (previously unmentioned in the main text). Specifically, we evaluated segmentation results using linear kernel CPD—which primarily detects changes in the mean—applied to the z-scored time-frequency representation of gyroscope signals, and compared them to those obtained using Gaussian kernel CPD on z-scored gyroscope data. Overall, both methods produced broadly similar segmentations. However, **wet dog shake (WDS) events were consistently oversegmented by the linear kernel CPD, whereas the Gaussian kernel CPD successfully identified them as individual segments**. This oversegmentation of WDS events by linear CPD led to an artificially reduced median segment duration. In contrast, the Gaussian kernel yielded a more balanced segmentation with interpretable median segment duration. **This result is now clearly mentioned in our revised manuscript, p. 4, lines 98–99.**

5. *Why does this loss function include the length of each segment, when, I believe, the total loss will therefore just have the length of the recording, which is a constant, so this term is unnecessary?*

We agree that the initial phrasing in the supplementary methods was potentially misleading. The function $c(.)$, which we referred to as a "cost function" for each segment, corresponds to a "homogeneity cost" reflecting the segment's internal homogeneity. This homogeneity cost is a Gaussian kernel-based rbf function whose first term $b - a$ indeed represents segment length:

$$c_{\text{Gaussian}}(x[a:b[) = (b-a) - \frac{1}{b-a}\sum_{s,t=a}^{b-1}\exp(-\nu \parallel x_s - x_t \parallel^2).$$

This term originates from the general formulation of kernel-based segmentation costs, appearing essentially as part of a normalization and scaling strategy for comparing segment similarity across varying lengths. However, as the reviewer correctly pointed out, since the sum of segment lengths always equals the total duration of the time series (a constant), this term does not contribute to the optimization objective and has no practical effect in our setting.

Importantly, **the total cost function contains an additional penalty term that increases with the number of segments** K, in line with standard formulations of change-point detection:

$$(\hat{\tau}_2, ..., \hat{\tau}_{\hat{K}}) = \arg\min_{(\tau_2,...,\tau_K),K} \sum_{k=1}^{K} c(x[\tau_k : \tau_{k+1}[) + \lambda K.$$

We have updated the corresponding subsection in the supplementary methods (p. 48, lines 1183–1200) for clarity.

6. *Why did they use the Viterbi filter over the Baum-Welch algorithm (which should give more robust*

results) to train their HMM?

In our context, **there are two main motivations for using the Viterbi algorithm instead of the Baum-Welch approach for inference in HMM**. First, we are primarily interested in finding the most probable sequence of hidden states for the given sequence of observation rather than parameter estimation through density estimation. Second, the Viterbi algorithm has the key advantage of having a lower computational cost than the Baum-Welch approach, with the complexity only increasing linearly with the size of the chain.

Reviewer 2

The study offers an entirely IMU-based approach for fine-grained, unsupervised parsing of rodent behavior (DISSeCT) that bypasses the usual computer-vision pipeline. Overall, the paper seems to be useful for behavior segmentation.

### Specific Comments

0. Please add background information on current unsupervised methods. How is this method different (in addition to just being applicable to IMU data).

We have expanded the discussion to provide a more comprehensive comparison between our pipeline and existing approaches, including both IMU-based and video-based methods (p. 16, lines 479–501).

1. Comparisons with other unsupervised methods

1a. In Keypoint-MoSeq, segmentation and clustering are integrated in a hierarchical Bayesian model that simultaneously infers where a behavioral "syllable" starts and ends, as well as which syllable it is. In the present paper, segmentation is done first via kernel-based change-point detection (Section "Pipeline architecture"), and then the extracted segments are clustered using a GMM. There is no direct demonstration of whether a fully joint approach would outperform or produce different motifs.

We fully agree with the reviewer that the conceptual framework underlying Keypoint-MoSeq (KpMS) could, in principle, be applied to inertial data for parsing behavioral motifs. **In the revised manuscript, we include a direct comparison between our pipeline and KpMS using our concurrent 3D pose data (Methods: p. 26, lines 807–819; Results: p. 11–12, lines 325–367; Fig. S12–13)**. Specifically, we evaluated and contrasted runtime and memory requirements (Tables S3–5). **Our results indicate that adapting the KpMS architecture to IMU data would entail a prohibitive computational cost**, rendering this approach unachievable without significantly compromising temporal resolution, which we think is a key strength of our approach. We therefore conclude that substantial methodological innovation is required to extend graphical model-based approaches to long-duration, high-resolution inertial recordings, a challenge that lies beyond the scope of the present study.

These points are now addressed in a new paragraph in the Discussion (p. 16, lines 502–512).

1b. VAME uses a temporal VAE on pose data to learn embeddings of short windows of motion, then clusters

those embeddings (sometimes with an HMM). Again, because DISSeCT does not embed data via a neural network, it avoids the potential complexity and resource overhead of a deep VAE, but it might lose out on the automatic feature learning and nonlinear features that VAME provides. Instead, the authors rely on manually crafted features (240 of them) and then do PCA + GMM. A big question is whether a more data-driven and nonlinear embedding (VAE) might capture often-subtle IMU patterns that the curated features do not.

We agree with the reviewer that automatic feature engineering is an important future direction, as originally briefly noted in the Discussion. In response to the reviewer's comment, **we now expand on this point more thoroughly (p. 17, lines 519–525), discussing the fact that VAE-based approaches rely on fixed-length window segmentation**. Alternative architectures such as transformers may offer greater flexibility in handling variable-length segments.

2. The current study validates cluster assignments by having an observer label N video snippets per cluster, and then compute a "consistency score" if a single label dominates (Figure 1F). While that shows approximate coherence, it is effectively a manual annotation approach. Other standard metrics in unsupervised learning include purity, normalized mutual information, or silhouette scores. Surprisingly, the authors do not report any such measures. From a machine learning perspective, the argument that "consistency score" suffices feels incomplete—particularly because they are claiming fully unsupervised segmentation that "largely aligns" with human-labeled behaviors. Reporting something like purity or ARI (adjusted Rand index) would make the approach more quantitatively comparable both internally (e.g. across hyperparameters) and to other frameworks (Keypoint MoSeq, VAME).

We thank the reviewer for the opportunity to clarify the rationale behind our labeling strategy. Purity, as defined in the paper introducing VAME (Luxem et al. 2022), was calculated based on a comparison with human annotations made at the single-frame level—meaning annotators implicitly performed both segmentation and classification. In that context, Purity quantified the agreement between model-generated and human-assigned labels over a single recording, using the following formulation:

$$Purity(U,V) = \frac{1}{N}\sum_{u \in U} \max_{v \in V} |u \cap v|$$

where U it the set of manually assigned labels u, V is the set of labels generated by VAME v and N is the number of frames in the video.

Importantly, Luxem et al. 2022 reported only 72% inter-annotator agreement across three human observers, emphasizing that **human annotation cannot be treated as an absolute ground truth**.

In our study, frame-level annotation was only used to qualitatively validate the performance of our change-point detection step (Fig. S2A). Rather than extending this to longer video recordings, **we adopted a segment-wise labeling strategy focused on ethological content**: by visualizing and labeling video snippets corresponding to individual CPD-derived segments, we could explore cluster content in depth—identifying both well-isolated behaviors and instances where distinct behaviors were grouped together in the same clusters—without conflating this analysis with the separate challenge of manual temporal segmentation. Whereas Luxem et al. 2022 annotated 6 minutes of data, our approach enabled manual labeling of 1 hour and 10 minutes of segments, covering nearly 14% of the dataset.

We note that our cluster-wise *consistency score* has a formulation similar to Purity, and we

have accordingly renamed it *purity score* in the manuscript:

$$\rho = \frac{1}{|S|} \max_{c \in C} |\{s \in S \mid label(s) = c\}| \,,$$

where S is a set of video snippets s associated with a given cluster, and C is the set of behavioral categories c.

In summary, **our labeling strategy and cluster-wise purity score are not intended to evaluate the similarity between the output of our pipeline and human frame-level annotations, but rather to assess the behavioral coherence of the GMM clusters with respect to meaningful, human-interpretable ethological categories**.

3. The GMM is used to derive 48 clusters (p5-6, Fig. S4B) I see they use the BIC across different numbers of clusters, but the text conflates "48 mixture components" with "48 clusters", and it's important to confirm whether each mixture component actually maps one-to-one to a single behavioral cluster. Especially since they note that some clusters are "sparse" (Fig. S7) or contain ambiguous segments. If multiple mixture components are effectively merged later (e.g. through the HMM or operator inspection), the text should explicitly state how the final cluster identities are consolidated.

In our framework, **we define *clusters* as groups of segments for which a single mixture component has the highest responsibility**. Consequently, the number of clusters corresponds to the number of mixture components (48 in our case) with no subsequent merging. To avoid confusion with the HMM terminology, we refer to the output of the HMM as *states*, not *clusters*. **We have revised the Methods section accordingly to clarify this (p. 23, lines 727–732).**

4. I'm also confused about the logic for using a categorical HMM only at the end (Section "Behavioral patterns can be unveiled...", p.10). The results show that this HMM can disambiguate context-specific segments that cluster similarly in GMM space. But why not incorporate the temporal constraints (i.e., HMM) into the initial segmentation process, akin to Keypoint-MoSeq or MoSeq? In other words, is the pipeline simpler or faster by strictly separating change-point detection, GMM clustering, and then HMM post-processing? The authors claim this keeps things modular, but I would be curious if an ablation test shows how final motif quality might differ if you skip the HMM or if you incorporate it jointly before segmentation.

Our architecture was indeed specifically designed to (1) efficiently process high-resolution IMU time series at a low computational cost and (2) provide a modular, adaptable framework. As outlined above and discussed on p. 16, lines 502–512, **adopting a joint segmentation and clustering strategy—such as that used in KpMS—would currently require severe downsampling of the IMU data to maintain feasible computation time and memory usage**. Instead, we chose to perform the segmentation and clustering steps separately. This allowed the use of a segmentation strategy based on Gaussian kernel change-point detection, a method more agnostic to the nature of changes that an HMM, which is intrinsically limited by the distribution chosen for emission.

This approach allows us to preserve the native high temporal resolution of IMU data, which (1) proved valuable in **capturing rapid behavioral patterns** such as grooming bouts and wet dog shakes, (2) **could be further exploited to isolate other behaviors with fine-grained, high-frequency signatures**—such as chewing, teeth grinding, or fur nibbling—that

the current pipeline does not yet individualize, and (3) is particularly advantageous for **future integration with electrophysiological recordings**.

In our framework, we introduce a final HMM layer that operates on segment cluster identities—thus remaining computationally lightweight—as a means to group segments into higher-order action sequences. This addition may help resolve certain kinematic ambiguities, as illustrated in Fig. S11 and discussed on p. 11, lines 311–324.

5. Many animal behaviors can be nested at multiple timescales (e.g., whisking vs. grooming vs. longer bouts of rearing). It is unclear whether a single, universal penalty λ captures them all, or if the user might need a multi-level approach for more complex data. This concern is not specific to DISSeCT: Keypoint MoSeq's HMM-based segmentation also implicitly fixes a timescale, and VAME's window-based embeddings have an assumed window size. None of these methods, as far as I know, truly adapt to multiple timescales in a single pass. On the practical side, additional guidelines (e.g., instructions on how to choose λ based on, say, user-defined expectations or frequency-domain analyses) would help experimentalists who do not want to rely solely on a published timescale from a different context.

We appreciate the reviewer's comment highlighting the challenge of capturing behaviors that unfold across multiple timescales. We fully agree that segmentation granularity is a central issue in behavioral time series analysis, and that a single, fixed timescale may not optimally capture all types of behavioral motifs.

While we have not yet systematically explored behavior-specific tuning of λ—and therefore cannot offer concrete guidelines at this stage—we agree that this is an important and promising direction. Although DISSeCT does not currently implement a multi-scale segmentation scheme, **its low computational cost makes it well-suited for scanning ranges of λ values**, enabling users to tailor temporal resolution to specific behavioral sequences or experimental objectives. Moreover, the iterative nature of the PELT algorithm—where the most prominent change in the signal is sequentially added to the set of retained change points—could potentially be leveraged to **retrospectively assess change points at multiple timescales from a single pass**. **Our revised discussion now briefly mentions the idea of leveraging the low computational cost of DISSeCT for multi-scale data analysis (p. 15, lines 456–459)**.

6. The entire pipeline is validated on single-animal data in controlled arenas, which is valuable from a proof-of-concept standpoint but sidesteps complexities of multi-animal interactions. DISSeCT's reliance on a single head-mounted IMU presumably makes it tricky to separate signals if multiple animals interact or physically collide. In Keypoint MoSeq or VAME (which are typically video-based pose estimators), you at least attempt multi-animal identity tracking, though that is itself non-trivial. The authors should at least have a discussion of this issue towards the end of the paper.

Video-based multi-animal tracking presents methodological challenges, particularly when animals partially overlap, leading to occasional identity swaps following close interactions. In contrast, **IMU recordings offer fully individualized and independent per-animal measurements**. Our Bluetooth-based wireless architecture (Pasquet et al. 2016) supports the simultaneous use of multiple IMUs without signal interference. However, **a key limitation of multi-animal IMU recordings is the lack of spatial context**—specifically, the absence of information about inter-animal distance and orientation. In principle, **this limitation could be addressed by integrating minimal video-derived cues** (e.g., from a single overhead camera)

into our pipeline. **This point is now discussed in the revised manuscript (p. 15–16, lines 472–478)**.

7. Keypoint-based pipelines sometimes show reduced performance on out-of-sample populations whose size, coat color, or posture deviates from the training distribution. In principle, an IMU-based approach may be somewhat more robust to superficial morphological differences (as inertial signals can still pick up speed, angle changes, etc.). However, if subtle morphological or gait differences shift the distribution of features significantly, a newly collected dataset might need re-segmentation parameters (especially the penalty λ) or a newly fitted GMM. As it stands, the paper does not mention cross-genotype validations or how DISSeCT might be retrained or adapted for genetically diverse cohorts. It would be helpful if the authors clarified whether the current pipeline can "auto-tune" itself (using BIC or cross-validation for number of clusters) when the population differs profoundly in mass, limb length, or baseline movement speed.

Indeed, keypoint-based pipelines are only valid for comparing groups of animals with similar size and overall body proportions. By contrast—and as the reviewer rightly noted—our IMU-based approach does not share this limitation, provided that sensor placement and orientation are consistent across groups.

As now clarified in the revised Discussion (p. 15, lines 446–447), we recommend re-running the pipeline whenever the experimental model or conditions change. In such cases, we advise using the BIC to select both the appropriate number of mixture components and the covariance matrix type. **We also emphasize how the probabilistic nature of Gaussian Mixture Models (GMMs) can be leveraged to compare models trained on different animal groups (p. 17–18, lines 540–551)**.

8. Overall, I think the pipeline is strong in capturing time-varying inertial signals and addresses a clear gap by providing a flexible, purely IMU-based segmentation approach. However, I would have liked a direct quantitative comparison with Keypoint MoSeq, VAME on a shared dataset, and with at least a standard cluster metric (e.g. purity, ARI, mutual information). Right now, the assessment hinges heavily on manual annotation of top-segment video snippets. That approach works, but it makes it harder to compare to existing frameworks or to interpret the "48 clusters" as truly distinct. From a machine learning perspective, I'd suggest adding standard unsupervised metrics, clarifying parameter sensitivity, and possibly discussing how a deeper (VAE-based) embedding might integrate with their kernel-based segmentation method. Otherwise, the method remains an interesting alternative to camera-based pose estimation, especially in situations where cameras aren't feasible or where inertial readings offer finer kinematic signals at minimal computational cost.

In response to the reviewer's comment, **we applied an unsupervised behavioral mapping pipeline to 3D pose data derived from our concurrent multi-view video recordings (Methods: p. 26, lines 807–819)**. To our knowledge, the current implementation of VAME only supports 2D pose data, whereas Keypoint-MoSeq (KpMS) has been validated on 3D pose. **We therefore selected KpMS and adjusted its stickiness parameter to match the median syllable duration to that obtained with our CPD-based approach**.

Compared to DISSeCT, KpMS yielded proportionally more "locomotion" syllables and fewer "idle", "grooming", "rearing", and "orienting" syllables, suggesting that **both methods tend to focus on different aspects of behavior**. Purity scores were more robust to randomized selection, pointing to fewer ethologically ambiguous cases. Notably, however, DISSeCT successfully isolated rhythmic behaviors (such as specific grooming subtypes and wet dog

shake events) that KpMS failed to capture under our conditions. The Adjusted Rand Index (ARI) between the two clusterings was 0.19, indicating limited structural overlap.

DISSeCT also showed a substantial computational advantage: despite the higher IMU sampling rate, it ran five times faster than the GPU-based KpMS implementation (excluding the cost of 3D pose estimation). **These observations are now detailed in a new Results sub-section (p. 11–12, lines 325–367) and illustrated in new supplementary figures (Figs. S12 and S13).**

As suggested by the reviewer, we now also discuss the potential of leveraging representation learning (e.g., VAE-based approaches) for feature extraction **(p. 17, lines 519–525)**. Our multimodal IMU-video dataset, which we will make publicly available, will—to our knowledge—be the first of its kind in rodents, enabling future benchmarking across pipelines.

Reviewer 3

The authors present a technique for quantitative behavioral phenotyping in rats that uses a head-mounted IMU to bypass the need for cameras and video-based tracking. The method is particularly appealing because it's possible experimenters already mounting drives or miniscopes on the head could simply add an IMU and get relatively comprehensive behavioral phenotyping "for free" (although the authors did not explicitly test their system as a component of an ephys or imaging system). The authors show that they can segment and cluster dozens of different rat behaviors given only a small set of head inertial measurements. They end with an experiment showing that their measurements and analyses are sufficient to detect levodopa-induced dyskinesias in a 6-OHDA model of PD.

I really liked this paper, and it was a joy to read. The authors communicate clearly and have made beautiful, informative figures. I think the method is exciting and impactful, and I look forward to seeing it published. However, I did have several questions, comments, and concerns that I think, if addressed, would help readers gain a better sense of the approach and its limits (and thus gain more trust in the method and its potential power).

Note, there are situations where using IMUs/DISSeCT would likely be far superior to video-based techniques. Not just in efficiency but in the ability to make measurements at all. Namely, when there are tons of occlusions in the environment (social groups, especially large groups; more natural arenas with lots of objects or housing; outside in the field, etc.) Obviously one can't do everything in a single paper, but if the authors wanted to elevate the work in the future, doing these types of experiments would really showcase the unique power of the technique.

Questions, comments, concerns:

1. I am confused about how the authors incorporated 3D pose data into their analyses. As best as I can tell, the poses are used only for visualizing example traces of their IMU clusters (and tracking the tail base velocity, which doesn't require tracking a full 3D pose). If just for visualization and qualitative validation, why do you need to track pose at all? This could have just been done with raw, untracked video.

2. I was expecting to see the 3D pose used in a different, quantitative way to validate their IMU clusters and defend their statements about the advantages of IMUs over (and as potential replacement for) video tracking approaches. Behavioral clustering from video is the current state-of-the-art, so it is important to compare directly to it.

Response to points 1 and 2. Our initial motivation for performing concurrent multi-view video recordings was twofold: (1) to enable detailed and accurate inspection of the ethological content of clusters, and (2) to create a publicly available dataset to support future comparisons across analysis pipelines. That said, we fully agree that a direct comparison with a 3D pose-based behavioral parsing method is relevant in the context of this manuscript. Accordingly, **we ran the Keypoint-MoSeq (KpMS) pipeline on our 3D pose data (Methods: p. 26, lines 807–819) and compared its output with that of our pipeline**. KpMS syllables were inspected using the same procedure applied to DISSeCT clusters. **The results of this comparison are briefly discussed in our response to Reviewer 2, point 8, are presented in a new Results sub-section (p. 11–12, lines 325–367) and illustrated in new figures (Figs. S12 and S13, Movies S4 and S5).**

3. Related to (2), you seem to find fewer clusters than previous studies quantifying rat behavior with 3D pose tracking. And potentially the clusters you find are less consistent (although cluster consistency is not often explicitly computed in other studies - I applaud the authors for doing this, though I do raise questions, below, about how they did it).

KpMS was configured so that the median duration of its syllable instances matched that of DISSeCT segments, as well as values reported in previous studies (Wiltschko et al. 2015; Weinreb et al. 2024). Under these conditions, **KpMS produced 26 relevant syllables** (defined as each covering at least 5% of the dataset), which is **consistent with the 30 syllables reported in the original KpMS study** using mouse 3D pose data (Weinreb et al. 2024). By comparison, our pipeline identified 48 clusters (Fig. S12B), **aligning closely with the 50 behavioral motifs described in the original VAME study** based on mouse 2D pose data (Luxem et al. 2022).

As the reviewer rightly noted—and to our knowledge—**no previous study has examined the ethological content of clusters as thoroughly as we do here**, making direct comparisons across methods challenging.

4. Aren't your consistency values going to be inflated and not representative of a cluster's quality overall if you are labeling only the top 200 posterior samples? I assume there are many more than 200 samples in a cluster, and that the samples with the lowest posterior probability are going to be the least consistent.

We agree that consistency scores (now referred to as *purity scores*; see our response to Reviewer 2, point 2) were inflated by selecting segments with the highest posterior probabilities for each cluster. To provide a more balanced assessment of cluster content, **we repeated the inspection procedure using a random selection of segments** (and KpMS syllable instances), as described in the revised Methods section **(p. 26–27, lines 826–853)**. As expected, purity scores decreased following randomization, though not uniformly across clusters and syllables (Fig. S12C–E) and across categories (Fig. S12F). Syllable-wise purity scores were less affected by this procedure than cluster-wise ones (Fig. S12F). When purity scores were computed at the level of the main behavioral categories, this difference was more nuanced, with DISSeCT achieving equivalent of higher scores for the "idle", "grooming" and "Orienting" categories (Fig. S12G).

5. More details on the consistency score, please. Specifically how are the no video / less than 7 frames segments accounted for - shouldn't they be integrated into your consistency? For instance, cluster 29 seems mostly consistent, but then you see that only a small fraction were valid behavior segments, so I would call that overall inconsistent, no?

A threshold of 7 frames (233 ms) was set for video snippet inspection, as **behavioral categorization became increasingly difficult below this duration**, as noted in the Methods **(p. 27, lines 840–841)**. Including all segments or syllable instances—regardless of duration—would have disproportionately inflated the "other" category, used when the observer was unable to determine the animal's behavioral state. Because clusters differ in their segment length distributions, they were differentially affected by this threshold. **Including shorter segments in this analysis would require a more time-consuming alternative**, such as incorporating contextual information before and after each segment or performing continuous frame-level annotation.

6. Can you provide some guidance on how to interpret the consistency values? To me they seem kind of low, but perhaps I don't have a good baseline. What do consistency scores look like for pose tracking clusters? Why not try this comparison if you have the data?

In the revised manuscript, **we provide a detailed comparison of purity scores obtained with KpMS and DISSeCT**. When considering the top 100 segments or syllable instances per cluster/syllable, cluster-wise and syllable-wise purity scores were broadly comparable, with over 60 % of clusters or syllables exceeding a score of 0.8 (Fig. S12G). KpMS scores were less affected by randomization than those from DISSeCT (Fig. S12G). However, as noted in our response to point 4, this difference was less pronounced when purity was computed at the level of behavioral categories, with DISSeCT slightly outperforming KpMS for the "idle" and "grooming" categories (Fig. S12F).

Our purity scores are conceptually related to the purity metric used in the VAME paper (Luxem et al. 2022), where human annotations at the single-frame level were compared with automatically assigned labels. A key difference is that in Luxem et al. 2022, purity was computed over a single 6-minute recording, during which the full behavioral repertoire may not have been expressed (e.g., grooming appears largely absent in the provided ethograms). In contrast, our purity scores were computed per cluster following inspection of representative segments spanning more than an hour of recording (see response to Reviewer 2, point 2). The values reported in Luxem et al. 2022 fall in the 0.75–0.80 range—a range achieved by 40 % of our clusters even under random segment selection. At the level of main behavioral categories, DISSeCT achieved scores in the 0.6–0.7 range, while KpMS scores ranged from 0.5 to 0.9 (Fig. S12F). **These results are discussed in the revised manuscript (p. 11–12, lines 336–343).**

As emphasized in both the manuscript (p. 12, lines 350–353) and our response to Reviewer 2, point 2, **absolute purity scores should be interpreted with caution**, as some nested behaviors—such as locomotion combined with orienting movements—can remain ambiguous even to human observers.

7. What happens if you cluster based on pose and then looking at distributions of head kinematics? Or overlay pose cluster ID onto your IMU S-UMAP?

We did not perform the first suggested analysis, as we can expect KpMS syllables to be associated to different head kinematic contents. **Given that our manuscript already characterizes the salient head kinematic signatures associated with different behaviors, we feel this additional analysis would be somewhat circular.**

The second suggestion—overlaying KpMS syllable IDs onto our embedding—poses a technical challenge: the features used for embedding are computed at the segment level after

change-point detection, whereas KpMS syllable boundaries do not necessarily align with those segment limits. **This results in complex cases where multiple KpMS syllables may overlap with a single segment, making such mapping ambiguous and difficult to interpret.**

8. Did you try an ARHMM directly on the head kinematic measurements? Would be too much to ask to implement, but mention of its possible advantages/disadvantages would be great.

The potential to extend graphical model-based approaches—such as AR-HMMs or KpMS—to IMU data is now discussed in the revised manuscript (p. 16, lines 502–512; see also our response to Reviewer 2, points 1a and 4). Based on our experience with KpMS runtimes on 3D pose data, **the main current limitation lies in the prohibitive computational and memory demands of applying such models to IMU data recorded at high sampling rates** (300 Hz). Achieving tractable runtimes would require substantial downsampling, which would undermine one of the key advantages of IMU recordings, i.e. their fine temporal resolution.

9. I think the supervised UMAP (S-UMAP) visualizations are a little misleading, as they give the false impression of there being super distinct clusters based on features alone (i.e., with an unsupervised UMAP) . It is particularly misleading because most everyone in this space is using unsupervised embeddings, so it produces this false sense that the IMU data is far superior. What about also including unsupervised UMAP in the main figure? This actually would be a nice tie-in to previous work, which would allow readers to better understand your study in the context of previous work.

We agree that the distinction between supervised and unsupervised embeddings should be made more explicit to avoid any misinterpretation about the separability of the clusters based on features alone. In response, **we have revised the main text to clearly emphasize that the embedding shown in the figure was obtained using a semi-supervised variant of UMAP, solely for visualization purposes (p. 5, lines 123–129)**. We also include fully unsupervised embeddings (UMAP and t-SNE) with superimposed cluster labels in Fig. S6.

Crucially, unlike some prior approaches that use low-dimensional embeddings for clustering or segmentation, **we employed semi-supervised UMAP solely as a visualization tool to jointly represent the feature space and the cluster identity assigned during a separate, fully unsupervised clustering step** (GMM over segment-wise features). Thus, the embedding had no influence on the clustering process itself.

We opted for this approach as it provides a compact and interpretable way to visualize both intra-cluster feature consistency and inter-cluster boundaries. Importantly, we verified that when cluster identities were randomly shuffled prior to semi-supervised UMAP, the resulting embedding was visually similar to the unsupervised version, indicating that the inclusion of labels did not impose artificial structure (Fig. S6).

10. Are there analyses you could do to better understand how the cluster IDs are influencing the S-UMAP space? What does it look like if you run GMM on a random matrix and then embed with S-UMAP? Or for a shuffled version of your data?

We agree that understanding the influence of cluster identities on the S-UMAP embedding is important. **As a control, we included in our analyses the case where cluster identities were randomly shuffled prior to embedding**. This is shown in Fig. S6, where the S-UMAP embedding with shuffled cluster labels closely matches the unsupervised UMAP result, confirming that when label information does not align with the structure in the data, the

supervised embedding does not artificially create separability.

As for the reviewer's question about applying a GMM to purely random input followed by S-UMAP, while we did not run this analysis, we expect the resulting embedding to reflect the imposed cluster labels from the GMM—even though these would be arbitrary and not reflect any underlying structure. This behavior is inherent to the S-UMAP algorithm, which partially aligns the embedding with the given labels. It underscores the importance of interpreting S-UMAP plots in the context of meaningful clustering and not as an independent test of feature separability.

To clarify this point, we have updated the manuscript (p. 5, lines 123–129) to explicitly state that S-UMAP is used purely for visualization purposes in our study, and not during the clustering itself. Its main role is to provide a compact view of how features and cluster identity relate in a low-dimensional space, not to validate cluster separability.

11. If the S-UMAP structure is primarily driven by cluster ID, what purpose do the S-UMAP visualizations serve that couldn't instead be communicated with a heatmap of the features, or equivalent?

While the use of S-UMAP does encourage separation between clusters based on cluster identity, the global geometry and fine-scale structure within each cluster are still largely driven by the underlying segment-level feature values. This is evident in Fig. 1B, where continuous variations in selected features (e.g., median pitch, Δ pitch, roll, azimuthal speed) reveal coherent gradients that are spatially organized across the embedding. These gradients reflect meaningful relationships in the data that are not captured by cluster identity alone.

In contrast to a heatmap, which requires a predefined ordering of clusters or segments and lacks spatial continuity, the 2D S-UMAP representation offers an interpretable, spatially coherent summary of how high-dimensional features vary both within and across clusters. This enables intuitive identification of clusters that share similar dynamics or occupy adjacent regions in the feature space, and helps visually validate the clustering outcome. Moreover, outlier segments excluded from the clustering still map coherently within this embedding, supporting the view that the embedding respects the intrinsic structure of the data.

Importantly, as emphasized in the revised main text **(p. 5, lines 123–129), S-UMAP was used solely for visualization purposes and was not involved in the clustering process itself, distinguishing our approach from prior embedding-driven clustering pipelines**.

12. For mixed grooming clusters and the like, kinematic ambiguities were mentioned as a possible cause. Can the authors reflect on whether they have evidence that the issues stem from the measurements and not the clustering approach? Because if it's the clustering approach, that means the problem could potentially be solved with future iterations or advances in the clustering, or with application of alternative existing clustering approaches (in a future study).

We believe that kinematic ambiguities could be addressed through two complementary strategies: (1) **incorporating minimal video-derived information into DISSeCT**, as discussed in the revised manuscript **(p. 15, lines 468–473)**, and (2) **optimizing feature selection and clustering resolution for specific behaviors (p. 15, lines 464–465)**.

For instance, some grooming-related clusters include segments where the head is pitched downward and exhibits rhythmic motion, yet these correspond to instances where the animal is actually sniffing or gnawing at the arena corners—particularly in a subset of sessions where a white sheet was placed along the inner wall to prevent reflections. These segments, labeled

Investigating corner in Table S6, could be distinguished by the absence of the characteristic crouched posture typically associated with grooming. In contrast, KpMS assigned such segments to immobility syllables, likely because the subtle orofacial movements involved were not captured by the video data.

Conversely, some grooming behaviors that appear similar in posture nonetheless differ in their head inertial signatures—differences that are not yet clearly distinguished by our pipeline. This is particularly true for *fur nibbling*, a grooming subtype characterized by a crouched posture resembling *body licking*, but with a static head and a high-frequency rhythmic signal detectable by the IMU. This behavior appears confined to a subset of segments within a single cluster (Fig. 5B). Its distinct gyroscopic spectral signature suggests that it could be better isolated in future studies—for example, by applying a secondary GMM to grooming segments using a dedicated set of features.

13. In your PD experiment analyses, sham is always going to be better fit across all time points, by virtue of the sham animals having been the ones used to fit the GMM. Have you tried fitting the GMMs to a subset of the sham? You could do this with a cross-validation approach (say, leave-one-out) and analyze each sham animal when it is in the "test" set, to better match how the PD condition is analyzed.

To address the concern regarding potential overfitting of the sham data, we implemented the suggested leave-one-out cross-validation procedure for evaluating the SUSM (Score Under Sham Model). Specifically, for each sham animal, we trained a Gaussian Mixture Model (GMM) using data from all other sham animals and computed the log-likelihood of segments from the left-out animal under this model. **This approach ensures that each sham animal is evaluated under a model it did not contribute to**, thereby aligning more closely with how PD and PD + L-DOPA animals are assessed.

For animals in the PD and PD + L-DOPA groups, we maintained the original approach of evaluating segments using a single GMM trained on all sham data, as this model serves as a normative reference for healthy behavior. **These methodological updates are now detailed in the Methods section (p. 28, lines 874–879), mentioned in the Results section (p. 13, lines 389–390) and the updated SUSM results are presented in revised Fig. 7C,D.**

Using this refined procedure, we confirmed a significant decrease in SUSM scores for the L-DOPA group compared to Sham animals, reinforcing the behavioral divergence introduced by dyskinesia. Notably, the difference between Sham and PD animals was no longer statistically significant under this leave-one-out approach, which is consistent with the more subtle behavioral alterations associated with the PD condition.

14. For the LID result, again it would be nice to know more about the interplay between the supervised and unsupervised components. Is the erroneous cluster identity over-accentuating the actual kinematic differences between the LID and sham data? There could be relatively small differences in the underlying features that lead to a change in hard cluster assignment to the sham-GMM - would this then produce misleadingly large differences in the S-UMAP embedding? What does it look like if you do unsupervised clustering alone?

We thank the reviewer for raising this important point regarding the interplay between supervised and unsupervised components in our analysis. To clarify, **the GMM used for clustering was trained exclusively on the Sham dataset**. This GMM was then applied to PD and PD + L-DOPA groups to assign cluster identities to their respective segments. **These**

predicted cluster labels were used as partial supervision when computing the S-UMAP embeddings—providing structure while still allowing the embedding to reflect continuous variation in kinematic features.

This semi-supervised approach was chosen specifically to enable the comparison of how shared behavioral components (as defined by the Sham-trained GMM) are kinematically altered across conditions. For instance, in Fig. 7D,E, we focused on cluster 0—one of the shared components—and observed systematic shifts in its kinematic expression between Sham, PD, and PD + L-DOPA groups. This illustrates how the method can sensitively capture nuanced motor changes within conserved behavioral motifs.

However, we acknowledge the reviewer's concern that applying the Sham GMM to L-DOPA segments may lead to imperfect or even misleading cluster assignments, particularly for motor patterns that are novel or substantially altered in this condition. Because S-UMAP uses these labels to guide the embedding, it may overstate the separation between groups if clusters are assigned inconsistently due to small changes in underlying features.

To address this possibility, **we also computed a fully unsupervised UMAP embedding (now added as Fig. S14), using only the kinematic features without any cluster labels**. As shown in this embedding, we observe the emergence of a distinct region in the PD + L-DOPA group that is not present in Sham or PD. This supports the idea that L-DOPA treatment induces novel movement patterns not captured by the Sham-trained GMM.

In summary, we retained S-UMAP as our main visualization tool because it is particularly effective at revealing subtle differences in the execution of shared behaviors. However, **we now include the unsupervised version in Fig. S14, and updated the main text accordingly (p. 13, lines 386–388), to provide a complementary view that better captures novel behaviors**. We believe the combination of both approaches gives a more complete picture of L-DOPA-induced motor changes.

15. Can the authors comment on why the PD phenotype (pre levodopa) was not obvious from the S-UMAP maps?

The absence of a clear distinctions in the S-UMAP embedding for PD mice prior to L-DOPA administration is primarily due to the subtle nature of the behavioral changes observed in this condition. Motor deficits resulting from unilateral 6-OHDA lesions in the medial forebrain bundle (MFB) are typically mild and often difficult to detect through the observation of spontaneous behavior in an open field. To reveal the underlying motor impairments, studies usually rely on more sensitive assays, such as motor performance or drug-induced behavioral tests.

As detailed in the revised manuscript **(p. 13, lines 405–407)**, PD mice in our dataset displayed prolonged immobility and a greater prevalence of slow, ipsiversive movements, consistent with previous findings (Carvalho et al. 2013; Slézia et al. 2023). In our pipeline, these behaviors are captured as a small number of long-duration segments. As a result, **their associated density in the UMAP space is relatively low, which limits their visual salience in the global embedding**.

However, **these behavioral differences are clearly reflected in cluster usage patterns** (Fig. 8A), where PD mice show a marked overrepresentation of two specific clusters—one corresponding to immobility, and the other to slow ipsiversive exploration. Thus, **while S-UMAP is valuable for visualizing feature structure and cluster identity jointly, it is**

inherently less sensitive to infrequent but behaviorally meaningful long-duration states.

16. I am curious about the 3 LID animals that look more like sham and whether there could be any sort of batch/cohort effect at play here. Were the sham animals run in a separate batch (e.g., different litter or a long time apart or a different experimenter or a different IMU installation point, etc)? Is there a chance, say, that something unrelated to LID is driving the observed difference? Example: the 9 animals with the clear LID effect are from a different litter, or were performed at different time of day, and just happened to become more lethargic or habituated more to the arena over time.

The experiments were conducted in two separate batches, three months apart: batch I included 2 PD and 2 SHAM mice, while batch II included 10 PD and 2 SHAM mice. Of the three LID animals that exhibited behavioral profiles more similar to SHAM controls, two were from batch I. **Therefore, we cannot entirely rule out a batch effect**.

However, the development of abnormal involuntary movements (LID) in 6-OHDA–lesioned mice depends on both the extent of dopaminergic denervation and the dose of L-DOPA administered (Francardo et al. 2011; Lundblad et al. 2004). In our protocol, 6-OHDA was injected into the MFB, followed by daily administration of L-DOPA at 6 mg/kg. **Under these conditions, previous studies report that approximately** 60 % **of mice develop LID** (Lundblad et al. 2004). In our cohort, this proportion reached 75 %, with 9 mice exhibiting a total LID score of 18.7 ± 3.1 at week 6. The remaining 3 mice—those showing low SUSM values under L-DOPA—displayed significantly lower LID scores (4.8 ± 3.7). In these same animals, striatal dopaminergic denervation was slightly less severe than in the other 9 lesioned mice (94.5 ± 1.2 % vs. 97.9 ± 1.6 %), which may partly account for their attenuated LID phenotype.

Overall, the variability observed in SUSM metrics are consistent with the one classically reported in LID severity—a variability whose causes remain unclear.

17. I would suggest an expanded discussion of possible limitations in the text. For example, the invasiveness of the IMU vs. video tracking; scalability of the procedure (requires a surgical procedure, whereas video tracking doesn't); reduced interpretability compared to pose tracking (you showed poses to aid with interpretability in the paper, after all). Just curious, have you ever tried to regress poses from inertial variables?

As suggested by the reviewer, **we have added a paragraph discussing the constraints and challenges associated with IMUs (p. 18, lines 552–565)**. While deriving head tilt and 3D head dynamics from IMU data is relatively straightforward (**Fayat2021Vestibular**), **we have not attempted to predict full-body poses from inertial signals**. **We anticipate that such an analysis would be limited by ambiguities in the mapping between head kinematics and body posture**. For instance, similar upward head pitch movements can occur with or without rearing, and leftward or rightward head rotations may involve body rotation or not.

Relatively minor:

1. Will their IMU fit on a mouse? Potential discussion point.

As detailed in the Methods section (p. 19, lines 599–601), **we used a** 1.85 g **wireless IMU for mouse recordings in the levodopa-induced dyskinesia experiment**. A wireless sensor was essential to prevent cable twisting due to the circling behavior characteristic of the PD + L-DOPA group. For rat recordings, we employed a 0.3 g wired IMU (p. 19, lines 579–580), which would also be perfectly suited for use in mice.

2. L69: "artifact-free" is a strong claim and can't possibly be true - can you just stick with the claim of "low noise"?

By describing the IMU signals as "artifact-free", we intended to highlight their superior noise characteristics relative to analog sensors and keypoint-based data. **As recommended by the reviewer, we have replaced "artifact-free" with "low-noise" in the revised manuscript (p. 3, line 68)**.

3. L112: "different shapes" - gives the impression these shapes can be arbitrary, but they still have to be Gaussian (just not spherical Gaussian).

We updated the sentence as follows: "*Unlike standard clustering algorithms such as K-means, GMMs can capture a broader variety of cluster shapes—not just hyperspherical ones—by modeling the underlying data distribution as a mixture of Gaussian components*" (p. 5, lines 114–116).

4. L116: BIC.. can also be used with K-means. K-means is, after all, just a special case of a GMM. Also, did you try a cross-likelihood analysis? BIC is notoriously unreliable.

While K-means can be viewed as a limiting case of a GMM with all cluster proportions assumed to be equal (clusters of same sizes on average) and all covariance matrices assumed to be equal as well as diagonal (clusters of the same spherical shape) with a unique variance term (isotropic structure), it does not define an explicit observed-data likelihood function. More precisely, **the link between K-means and GMM can only be made at the inference level**. Considering these assumptions, minimising the intra-class inertia is indeed equivalent to maximising the complete joint log-likelihood over the data and the allocation vectors. However, we would like to emphasise that maximising such complete joint log likelihood leads to estimators which are biased and which are not the maximum likelihood estimators (MLE) which are required to compute the BIC criterion. Indeed, the BIC criterion comes from a Laplace approximation and therefore needs to be evaluated only at the MLE.

We acknowledge that BIC has limitations—particularly with small data sets or when none of the candidate models fully capture the true data distribution (model misspecification). **Nonetheless, BIC provided a practical and well-established way to balance model fit and complexity in our setting**.

Moreover, **given the small number of animals (n=4 rats), we opted against cross-validated likelihood approaches**, as they would have severely reduced training data per fold and introduced high variance. For these reasons, and in line with common practice for GMM selection, we retained BIC as our model selection criterion.

5. Missing citation and discussion of Ouyang et al.. 2024 Neuron, which uses IMUs to segment behaviors (although not as extensively as your paper does).

The paper suggested by the reviewer, like several other studies in rodents, employs convolutional neural networks to automate behavioral scoring based on fixed-length window segmentation. **In the revised manuscript, we have incorporated this reference in our brief overview of IMU-based supervised behavioral classification approaches (p. 16, lines 479–480)**.

6. I found myself wanting to know earlier on in the paper (say, in the Introduction), exactly how many / what type of IMUs were being used.

We updated the introduction as follows **(p. 3, line 71)**: *"We ran our pipeline on head inertial recordings obtained from freely moving rodents* ***using both wired and wireless 6-axis digital IMUs****, leveraging the fact that these animals rely heavily on head movements to explore their environment."*

7. No citation of Fig. 2D1 or Fig. 2D2 in the text.

Fixed **(p. 7, lines 191–192)**.

8. L186 paragraph - Found this paragraph confusing, it's written as if you've already described the segmentation of different locomotion phases (which I interpreted to mean different components of the gait cycle). If by "phases" you mean periods when the animal is locomoting, then I suggest a different term to avoid ambiguity. Also, I personally find the statement about (paraphrasing) "because there is no location information one shouldn't necessarily expect to identify locomotion segments" a weak straw man - I don't think anyone would expect the head to be perfectly still during locomotion.

In the revised manuscript, **we have simplified the text by removing this paragraph and introducing the analysis of locomotion clusters by the sentence**: *"To understand what caused the clustering algorithm to isolate locomotor activity, we examined the head kinematic features associated with the three "locomotion" clusters highlighted in Fig. 3A.* **(p. 8, lines 209–210)**.

9. L358 - I don't think you know whether you've captured the "the full repertoire" of head movements - there could be many behaviors you're not observing, especially when just using an unnatural open field box. Do you need to claim "the full"?

In the revised manuscript, **we have rewritten the sentence as follows**: *"A visually informative overview depicting the* ***corresponding*** *repertoire of such movements was obtained [...]"* **(p. 7, line 188)**.

10. L397 - Re: clustering after dimensionality reduction to 2d, "its drastic dimension reduction step prior to clustering likely diminishes its discriminative power [70]". "Likely" is an overstatement. Something like "potentially" would be better. The study you cite is on drosophila behavioral clustering, and you've made no direct comparisons to clustering in 2D (although you could...).

We replaced "likely" with "potentially" **(p. 16, line 494)**.

11. L427 - "DISSeCT also meets the requirements of a robust deep phenotyping tool." I think it is fine to say it holds promise, but I wouldn't say you've "met the requirements" yet. First, what are the requirements? How did you explicitly test for robustness? It didn't detect differences in all animals, so that seems to be a knock against "robustness." Also, the detected changes in PD (pre-L-DOPA) were relatively small, despite the 6-OHDA model being quite a strong perturbation, you did not test it in subtler PD models, did not detect a positive L-DOPA rescue of a PD phenotype, etc. I'm not saying you needed to have done all of this, but the claim you make here should reflect how there is probably more needed to "meet the requirements".

We have replaced this sentence with: *"our pipeline offers a promising new solution for deep behavioral phenotyping"* **(p. 18, line 548)**.

We hope these revisions satisfactorily address the reviewers' concerns. We remain grateful for the opportunity to improve our work.

References

Luxem, Kevin et al. (2022). "Identifying behavioral structure from deep variational embeddings of animal motion". In: *Communications Biology* 5.1. DOI: 10.1038/s42003-022-04080-7.

Pasquet, Matthieu O et al. (2016). "Wireless inertial measurement of head kinematics in freely-moving rats". In: *Scientific reports* 6.1, p. 35689. DOI: 10.1038/srep35689.

Wiltschko, Alexander B. et al. (2015). "Mapping Sub-Second Structure in Mouse Behavior". In: *Neuron* 88.6, pp. 1121–1135. DOI: 10.1016/j.neuron.2015.11.031.

Weinreb, Caleb et al. (2024). "Keypoint-MoSeq: parsing behavior by linking point tracking to pose dynamics". In: *Nature Methods* 21, pp. 1329–1339. DOI: 10.1038/s41592-024-02318-2.

Carvalho, Miguel M. et al. (2013). "Behavioral characterization of the 6-hydroxidopamine model of Parkinson's disease and pharmacological rescuing of non-motor deficits". In: *Molecular Neurodegeneration* 8.1, pp. 12–14. DOI: 10.1186/1750-1326-8-14.

Slézia, Andrea et al. (2023). "Behavioral, neural and ultrastructural alterations in a graded-dose 6-OHDA mouse model of early-stage Parkinson's disease". In: *Scientific Reports* 13.1, pp. 1–17. DOI: 10.1038/s41598-023-46576-0.

Francardo, Veronica et al. (2011). "Impact of the lesion procedure on the profiles of motor impairment and molecular responsiveness to L-DOPA in the 6-hydroxydopamine mouse model of Parkinson's disease". In: *Neurobiology of Disease* 42.3, pp. 327–340. DOI: 10.1016/j.nbd.2011.01.024.

Lundblad, M. et al. (2004). "A model of L-DOPA-induced dyskinesia in 6-hydroxydopamine lesioned mice: Relation to motor and cellular parameters of nigrostriatal function". In: *Neurobiology of Disease* 16.1, pp. 110–123. DOI: 10.1016/j.nbd.2004.01.007.
