# Supplementary material for: DISSeCT: An unsupervised framework for high-resolution mapping of rodent behavior using inertial sensors
Source: PLoS Biol. 2025 Oct 9;23(10):e3003431. doi: 10.1371/journal.pbio.3003431 (PMC12527166; doi:10.1371/journal.pbio.3003431)
Supplement: Attachment — Submitted filename: Fayat_et_al_rebuttal.pdf [file pbio.3003431.s030.pdf]

DISSeCT: an unsupervised analytical framework for high-resolution rodent behavior mapping using inertial sensors

PBIOLOGY-D-24-03343R1

Romain Fayat[1], Marie Sarraudy[1], Clément Léna[1], Daniela Popa[1], Pierre Latouche[2,3] and Guillaume P. Dugué[1*]

[1]Neurophysiology of Brain Circuits, Institut de Biologie de l'École Normale Supérieure (IBENS), École Normale Supérieure, CNRS, INSERM, Université PSL, 75005 Paris, France
[2]Université Clermont Auvergne, CNRS, Laboratoire LMBP, UMR 6620, 63178X Aubière, France
[3]Institut Universitaire de France (IUF)

We are pleased to learn that our manuscript is on track for formal acceptance. We would like to express our gratitude to you and the reviewers for your careful evaluation and constructive feedback. In this final revision, we have addressed the remaining points as follows:

Editorial requirements

We suggest the following alternative title for your study, to highlight your method's name and maintain accessibility for our broad audience: "DISSeCT is an analytical framework for high-resolution rodent behavior mapping using inertial sensors"

We propose the following title: "DISSeCT: an unsupervised analytical framework for high-resolution rodent behavior mapping using inertial sensors".

Competing interests

The first character in the word "the" is missing from your competing interest statement.

This was corrected in the online submission system.

Ethics

The Ethics statement needs to be the first subheading in the Material & Methods section. It must include the full name of the IACUC/ethics committee that reviewed and approved the animal care and use, as well as the protocol/permit/project license number.

The Ethics statement was pasted to the Material & Methods section, and edited as follows: *This project was reviewed by the "Charles Darwin" Ethical Review Board (CEEA-005) and approved*

*Corresponding author: guillaume.dugue@cnrs.fr

by the department responsible for overseeing the use of animals for scientific purposes within the French Ministry of Higher Education, Research, and Innovation (AFiS unit). Approval was granted under project license number APAFIS #29793-2021021217521 92 v3.

Data

Your data availability statement says that the data will be made accessible upon acceptance of the manuscript. As your paper is likely to be formally accepted, please make the data accessible now and provide a link.

A link to our dataset (hosted on ebrains.eu) has been added in a new "Data availability" section, following the "Code availability" section:
The IMU and 3D pose datasets supporting this study are publicly available at: https://doi.org/10.25493/8ATR-V87.

In addition, please note that we require the numerical values in a supplementary file or as a permanent DOI'd deposition for the following figures: 3E, 7C.

NOTE: the numerical data provided should include all replicates AND the way in which the plotted mean and errors were derived (it should not present only the mean/average values).

Please cite the location of the data clearly in all relevant main and supplementary Figure legends, e.g. "The data underlying this Figure can be found in S1 Data" or "The data underlying this Figure can be found in https://doi.org/10.5281/zenodo.XXXXX"

The data underlying Fig 3E and Fig 7C are now provided as supporting data files (S1_Data.csv and S2_Data.csv). These files are referenced in the legends of Fig 3 and Fig 7, and their contents are described in the "Supporting information" section of the manuscript, which contains only the legends of the supporting material.

If you are reporting experiments where $n \leq 5$, please plot each individual data point.

In Fig. 7C, the SHAM group includes 4 animals. We are concerned that adding individual data points may reduce the readability of the graph. Please advise.

Supplementary files (e.g., excel). Please ensure that all data files are uploaded as 'Supporting Information' and are invariably referred to (in the manuscript, figure legends, and the Description field when uploading your files) using the following format verbatim: S1 Data, S2 Data, etc. Multiple panels of a single or even several figures can be included as multiple sheets in one excel file that is saved using exactly the following convention: S1_Data.xlsx (using an underscore).

Supplementary files were uploaded separately following the above guidelines.

Code availability

Thank you for providing the underlying code in GitHub. However, because Github depositions can be readily changed or deleted, please make a permanent DOI'd copy (e.g. in Zenodo) and provide this URL in the manuscript and Data Availability Statement.

We now provide links to Zenodo copies of the repositories of DISSeCT and INSpECT in the "Code availability" section.

Additional editorial comments

As you address these items, please take this last chance to review your reference list to ensure that it is complete and correct. If you have cited papers that have been retracted, please include the rationale for doing so in the manuscript text, or remove these references and replace them with relevant current references. Any changes to the reference list should be mentioned in the cover letter that accompanies your revised manuscript.

A final verification of our reference list confirmed that none of the cited papers has been retracted. In addition, three bioRxiv preprints have since appeared in peer-reviewed journals, and we have updated the references accordingly. Please note that we have added a citation (number 89) in the modified discussion on model generalizability (p. 11).

Reviewer remarks

Reviewer #3: I have reviewed the revised manuscript and the authors' responses to all reviewers. The authors were impressively responsive to reviewer feedback and have performed many new analyses that I think satisfactorily address all major reviewer concerns.

I have a few additional comments:

Re: Reviewer 2, comment 7. The authors added to the discussion section a recommendation that the pipeline should be rerun whenever the experimental model and conditions change. However, there are clear disadvantages to this that are also important to mention in the discussion (along with communicating why it might be important to address these issues in future work). Also, with pose data, if the data from two experiments is not drastically different, it is common to combined and embed the datasets together. I think it is worth mentioning under which conditions this might work for IMU data and your model, rather than simply saying researchers should always train a new model.

We agree that our initial recommendation to systematically rerun the pipeline may have appeared overly blunt. In response to the reviewer's comments, we have revised and expanded the discussion (p. 10–11) to clarify our position. Specifically, we now emphasize that:

- Provided sensor placement is reproducible, model generalizability can reasonably be assumed under similar conditions, as is the case for pose-based models.
- Generalizability is inevitably challenged when experimental conditions change.
- Our framework offers tools to help determine whether model retraining is necessary, while leaving the final decision in the researcher's hands.
- We outline a possible strategy for improving generalizability through the use of a global reference dataset.
- The computational and practical burden of rerunning DISSeCT is mitigated in several ways.

Re: Reviewer 3, comments 9-10. I remain unconvinced of S-UMAP utility. The authors state that S-UMAP enhances interpretability or "more clearly reveals the internal structure" or clusters or provides a more

interpretable "intra-cluster feature consistency and inter-cluster boundaries", but just because something looks nicer doesn't mean it's more accurate and reflective of underlying trends. What is the quantitative evidence that clusters being larger or smaller in S-UMAP reflects the underlying variance of those clusters. That the distance between S-UMAP clusters is indeed indicative of cluster similarity? Much of this could be imposed or influenced in unmeaningful ways by the supervision process.

However, I do appreciate that the authors have in their revision now used unsupervised UMAP where it is more essential to do so, to support their more essential claims related to PD.

We agree with the reviewer that visual appearance alone is insufficient to demonstrate the interpretability of S-UMAP, which depends on whether it preserves meaningful information about cluster size and separation. To address this quantitatively, we compared cluster spread and inter-cluster distances in the original PC space (where GMM clustering was performed) with those in the UMAP and S-UMAP embedding spaces (computed from z-scored features).

Cluster spread in the PC space was quantified as the trace of each cluster's covariance matrix (i.e., the total variance across all PCs), while spread in the embedding spaces was measured as the average Euclidean distance of cluster members from their centroid. We found that the correlation between these measures was not diminished in S-UMAP relative to UMAP, and was in fact slightly higher for S-UMAP (new panel B in S6 Fig). **This supports the conclusion that S-UMAP preserves the relationship between cluster variance in the PC space and apparent cluster size in the embedding.**

We performed a similar analysis for inter-cluster relationships by comparing pairwise distances between cluster centroids in the PC space with those in the UMAP and S-UMAP embeddings. Here too, centroid distances in S-UMAP remained clearly correlated with those in the PC space, albeit slightly less strongly than in UMAP (new panel C in S6 Fig). **This indicates that S-UMAP largely preserves inter-cluster distance information while offering improved visualization of the GMM results.**

The main text was re-written as follows: *"We then applied semi-supervised UMAP (S-UMAP) to project segments into a two-dimensional embedding of the standardized feature space, guided by the cluster identities obtained from the GMM. This embedding was not used for clustering, but to improve the readability of the GMM output by providing a clear visualization that preserves feature gradients (Fig 1B and S6A Fig). Importantly, despite the supervision, the embedding retained meaningful information about cluster variance and inter-cluster relationships (S6B,C Fig)."*
